# Characterization of the Small RNA Transcriptome of the Diatom, *Thalassiosira pseudonana*

**DOI:** 10.1371/journal.pone.0022870

**Published:** 2011-08-12

**Authors:** Trina M. Norden-Krichmar, Andrew E. Allen, Terry Gaasterland, Mark Hildebrand

**Affiliations:** 1 Scripps Institution of Oceanography, University of California San Diego, La Jolla, California, United States of America; 2 J. Craig Venter Institute, San Diego, California, United States of America; Miami University, United States of America

## Abstract

This study presents the first characterization of endogenous small RNAs in a diatom, *Thalassiosira pseudonana*. Small RNAs act as transcriptional and translational regulators, controlling specific target genes involved in various cellular functions. Diatoms are unicellular photosynthetic organisms that play major roles in environmental processes, such as food webs and global carbon fixation. Small RNA cDNA libraries were constructed for exponentially growing *T. pseudonana*, and then subjected to highly parallel pyrosequencing (454) and sequencing-by-ligation (Applied Biosystems SOLiD). From the computational analysis of approximately 300,000 sequences in the 454 library and over 17 million sequences in the SOLiD libraries, there exists evidence of a core set of small RNA genes including: novel microRNAs, repeat-associated short interfering RNAs, and endogenous short interfering RNAs. The diatom genome contains elements similar to plant small RNA systems, such as the RNAi machinery, a high percentage of short interfering RNAs originating from protein-coding and repetitive regions of the genome, and putative binding sites of the small RNAs occurring primarily in the coding section of the predicted targets. The characterization of the small RNA transcriptome of *T. pseudonana* establishes the possibility of a wide range of gene regulatory mechanisms in diatoms.

## Introduction

Diatoms are unicellular photosynthetic phytoplankton of global importance. They are responsible for 20% of global carbon fixation [1], [2], and as such not only provide a major source of carbon for food webs, but also are key players in atmospheric carbon cycling and its attendant environmental issues. Diatoms are in the heterokont class of microalgae, and have a distinct evolutionary history relative to land plants and red, green, and glaucophyte algae in that they are the result of a secondary endosymbiotic event, whereby a free living heterotroph acquired a plastid through enslavement of a red or green algae [3], [4]. This leads to a unique genetic complement in diatoms [5] and, by inference, potentially unique gene expression control mechanisms. In addition, the ocean environment is subject to dynamic changes that frequently occur on short time scales, and organisms in this environment must have gene expression control mechanisms to enable rapid adaptation to these changes.

Complete genome sequences have now been reported for two diatoms, *Thalassiosira pseudonana* [3] and *Phaeodactylum tricornutum* [6]. Both species were selected for their small genome sizes (32.4 Megabases (Mb) and 27.4 Mb, respectively). These two diatom genomes contain between 10,000 and 14,000 genes [3], [6], [7], [8], of which only around 50% of the genes can be assigned a putative function based on current experimental knowledge, and about 35% are specific for each diatom [6], [9].

Small non-coding RNA genes have been found in numerous organisms where they act as transcriptional and translational regulators of gene expression. Their ability to silence specific genes affects a wide range of biological functions, ranging from gene regulation during embryological development and cell differentiation, to genome rearrangement [10]. Of the classes of small RNAs, the microRNA (miRNA) family is the most extensively characterized. MicroRNAs are estimated to occur at a frequency of approximately 0.5–1.5% of the total genes in the genome of an organism [11], and it is estimated that 20 to 30% of human genes are regulated by miRNA [12]. Besides microRNAs, the remaining types of small RNAs may be grouped together collectively as endogenous short interfering RNAs (siRNA). Endogenous siRNAs have been found in multicellular and unicellular plants and animals [13]. The common characteristic of siRNA genes is that their biogenesis involves double-stranded RNA, without a hairpin precursor, which is in contrast to miRNAs [14]. The siRNA types of relevance to this study include repeated-associated siRNAs (rasiRNAs) and natural antisense siRNA.

While most research on eukaryotic small RNA to date has focused on multicellular plants and animals, there have recently been studies in unicellular eukaryotes. In particular, several types of small RNAs were reported in the unicellular green algae *Chlamydomonas reinhardtii*, following 454 sequencing of a small cDNA library [15], [16], and evidence of miRNAs has also been recently found in the heterokont brown algae *Ectocarpus siliculosus* [17] and the red algae Porphyra yezoensis [18]. Overall, these studies identified miRNAs, phased siRNAs, tasiRNAs, and nat-siRNAs. All of the miRNAs found that had the characteristic hairpin structure precursor, were novel and did not exhibit sequence identity with known plant and animal miRNAs. These results are significant, since they imply that deep sequencing might be the key to discovering miRNAs in organisms that have not yet been studied extensively for small RNAs, or in which specific miRNAs are not exceptionally highly represented. Highly expressed plant and animal small RNAs were initially characterized using traditional cloning approaches [19], [20], [21]. Re-examination of these organisms by deep sequencing approaches has revealed a larger population that includes miRNAs expressed at lower levels [22]. miRNAs with lower expression levels are generally not conserved between organisms, suggesting that they play specialized roles [22].

Applied Biosystems (ABI) SOLiD next-generation sequencing represents an emerging technology that may aid in the discovery of small RNAs. The SOLiD (Supported Oligonucleotide Ligation and Detection) platform utilizes a sequencing-by-ligation method, which involves iterations of hybridization and ligation, on a glass slide support, using probes labeled with four different fluorescent dyes [23], [24]. Each dye encodes a two-nucleotide pair, generating sequence data represented in “colorspace” format, rather than in nucleotide “base space” data format. The promise of resequencing applications and transcriptomic analyses has brought this next generation sequencing technology to the forefront [23], [25]. To date, there have been only a small number of published studies using SOLiD for identifying miRNAs in standard model organisms, such as human, rat, and Arabidopsis [26], [27], [28], [29], and no studies have been reported on a nonstandard model organism, such as the diatom.

In this study, we applied both 454 and SOLiD deep sequencing approaches to characterize classes of small RNAs from the diatom *Thalassiosira pseudonana*, determine their similarity to plant and animal small RNAs, examine their genomic distribution, and identify potential target mRNAs for regulation. Comparison of both approaches (which included two different sample preparation methods and biological and technical replicates) was done to obtain a broader-based look at the small RNA population, with 454 providing accurate evaluation of small RNA lengths and terminal nucleotides, and SOLiD providing deep coverage. For SOLiD data analysis, the standard ABI SOLiD data processing pipeline includes a step whereby the data is filtered by a comparison to the Sanger miRBase database of known miRNAs ([http://microrna.sanger.ac.uk/]) [30]. Because the majority of miRNAs in the diatom may be novel, as was found for *Chlamydomonas* miRNAs [15], [16], filtering by the known Sanger miRBase may have undesirable effects. Therefore, this study also reports the development of a new methodology to process SOLiD data to extract the entire small RNA population, which can then be examined to identify and predict novel miRNAs and endogenous siRNAs.

## Materials and Methods

### Experimental Methods

#### Cell culture

*Thalassiosira pseudonana* strain CCMP1335 was obtained from the Provasoli-Guillard National Center for Culture of Marine Phytoplankton, Bigelow Laboratory for Ocean Sciences (West Boothbay Harbor, ME, USA), and maintained in artificial seawater (ASW) medium [31], supplemented with biotin and vitamin B_12_, each at 1 ng·L^−1^. Cultures were magnetically stirred and aerated using sterile techniques. Two separate cell cultures were maintained at 18°C–20°C in continuous light at an intensity of 150 µmol photons · m^−2^ · s^−1^. One *T. pseudonana* exponential growth culture sample (Tp-EF) was grown in an 8-liter glass bottle of ASW to a density of 1.8×10^6^ cells · ml^−1^ and the other (Tp_EC) was grown similarly to a density of 1.14×10^6^ cells · ml^−1^. The Tp-EF cell culture sample was used to construct both a small RNA cDNA library for 454 pyrosequencing and a cDNA library for SOLiD sequencing. The Tp-EC sample was used to construct a biological replicate small RNA cDNA library for SOLiD sequencing.

#### Small RNA cDNA library construction for 454 library

Total RNA was extracted with TriReagent (Sigma) as previously described [32]. Following total RNA extraction, PEG/NaCl precipitation was performed to separate low and high molecular weight RNAs in order to generate a small RNA library suitable for deep sequencing [33].

**Figure 1:**
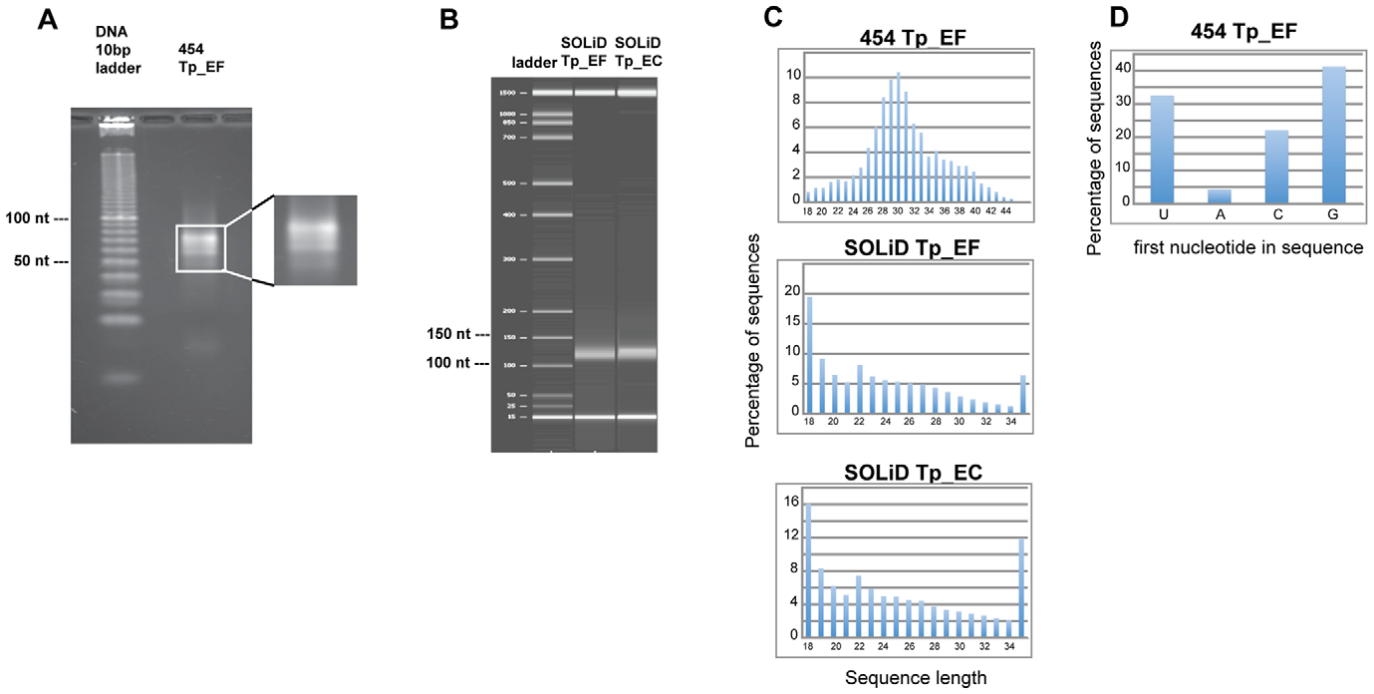
General characteristics of the small RNA libraries. **1A.** ***T. pseudonana*** **preparatory gel showing presence of small RNA bands after library construction and amplification, prior to 454 sequencing.** Agarose gel electrophoretic separation of final amplified small RNA products. Right, zoomed gel detail highlights the presence of several predominant bands and size classes. **1B.** ***T. pseudonana*** **Agilent gel showing presence of small RNA band after library construction and amplification, prior to SOLiD sequencing.** **1C - Length distribution of** ***T. pseudonana*** **small RNA candidate sequences.** Length distribution of unique sequences was calculated after removal of RNA degradation products and alignment with the *T. pseudonana* genome. **1D - Nucleotide frequency at the 5′ end of the small RNA candidate sequences.** Nucleotide frequency of unique sequences was tabulated after removal of RNA degradation products and alignment with the *T. pseudonana* genome.

Subsequent processing included size-selection using gel electrophoresis and ligation of specific linkers. MicroRNAs and small RNAs that are generated by an RNase III mechanism have 5′P and 3′OH terminal groups, which is the opposite of mRNA degradation products. This feature enables sequential ligation of appropriate linkers specifically to the small RNAs. Linkers successfully used in several other laboratories [20], [21] were used here. The 5′ linker (called “Nelson” [20], [21], ATCGTAGGCACCTGAAA), has a 3′ hydroxyl group, and was ligated to RNAs with a 5′ phosphate using T4 RNA ligase. The ligation product was size-selected and purified using denaturing PAGE as described [33]. The 5′ end of the 3′ linker (called “Modban”, ACTGTAGGCACCATCAAT), contains a 5′ phosphate that enables ligation to a 3′OH group, and a 3′ di-deoxyC group that acts as a block to prevent further ligation. Small RNAs generated from the protocol only exist in the configuration: Nelson linker, small RNA, Modban linker. Following purification of the second ligation product, the ligated small RNAs were subjected to RT-PCR with Superscript III RT (Invitrogen), using primers corresponding to the linker sequences. A 4% Metaphor agarose (Lonza) gel purification was used to isolate amplified small RNAs of the correct size (Figure 1A).

TOPO cloning (TOPO TA for Sequencng kit, Invitrogen) of the purified small cDNA fraction was performed, and approximately 50 transformants were grown for DNA preparation using a Qiagen QIAprep-spin Miniprep kit, and sequenced to confirm the quality of the library. Briefly, the purified PCR product was used to transform One Shot TOP10 Competent cells, and plated onto LB-ampicillin plates. The plates were incubated overnight at 37°C. Colonies were picked and placed into tubes containing 5 ml of 2XYT media and 5 µl of 50 mg/ml ampicillin. The tubes were shaken overnight at 37°C. Cloned DNAs were sequenced by the Sanger method using a service provided by SeqXcel (San Diego, CA).

After verification of the quality of the TOPO library, amplified cDNA material was quantified on an Agilent Bioanalyzer and approximately 160 ng of material was prepared for one half plate of 454 FLX sequencing [34] at the J. Craig Venter Institute (JCVI) in Rockville, MD.

#### Small RNA cDNA library construction for SOLiD libraries

Total RNA from *T. pseudonana* cell cultures was extracted with TriReagent (Sigma) as previously described [32]. To enrich for the ∼18–40 nt small RNA fraction, the total RNA samples were treated with the flashPAGE fractionator (Ambion #AM13100) and flashPAGE Clean-Up Kit (Ambion #AM12200). The small RNA enriched samples were then processed according to the SOLiD Small RNA Expression Kit protocol (Applied Biosystems, Life Technologies, Carlsbad, CA).

The first step of the SOLiD Small RNA Expression Kit protocol involved a ligation step with Adapter Mix A using RNA ligase, which requires an RNA with a 5′ phosphate and a 3′ hydroxyl group, as is characteristic of small RNAs. Adapter Mix A is used for SOLiD sequencing starting from the 5′ ends of the small RNA.

cDNA from each culture was barcoded by amplifying the library with a particular PCR primer set provided in the kit, where each primer set differed by a known 6-nucleotide sequence. Therefore, after sequencing, the samples could be identified and sorted into barcoded condition sequences. The barcode numbering scheme was as follows: Tp_EF was barcoded G00032, and Tp_EC was barcoded G31013.

The quality and quantity of the samples was verified on the Agilent Bioanalyzer (Figure 1B). Approximately 200 ng of each condition and adapter mix was prepared for Applied Biosystems SOLiD next generation high throughput sequencing at JCVI. Duplicates of each sample were run on two quadrants of the slide. That is, the first quadrant (A1) and second quadrant (A2) both contained a sample of the Adapter A mix preparation.

### Computational Methods

#### Computational search for RNA interference (RNAi) machinery in diatoms

The capacity of *Thalassiosira pseudonana* to employ RNAi-related mechanisms was evaluated bioinformatically. To assess sequence similarity to RNAi-related genes, the filtered gene model predicted transcripts for *T. pseudonana* was downloaded from the JGI website. Using the list of proteins in Text S1, a FASTA file was created that contained these protein sequences of interest from the UniProt site ([http://www.pir.uniprot.org/]). A BLASTp alignment [35] was performed between the gene models and the test set of protein sequences. The results were sorted by alignment length and E-value significance.

For evidence of pattern/motif similarity, the motifs lists in Text S1 were used to create a test set of Pfam identifiers from the Pfam database ([http://pfam.sanger.ac.uk/]). The Hidden Markov Model for Pfam software, hmmpfam, was used to search for the Pfam motifs in the filtered gene model predicted transcripts for *T. pseudonana*. Gene models containing matches to the domains of interest with appropriate HMM cutoffs were manually examined for completeness and, if necessary, extended. The extended gene models were then subjected to an expanded search.

Finally, a keyword search was performed using the following steps. The filtered gene model predicted transcripts for *T. pseudonana* were aligned with the BLAST program against the proteins in the non-redundant database, “nr” ([http://www.ncbi.nlm.nih.gov/]). The top match for each gene model was chosen as the functional description. The description text for each match was then searched for the presence of the protein and motif names listed in Text S1.

#### Data Files

Computational analysis of the small RNA sequence data was performed with the *Thalassiosira pseudonana* genome, version 3.0 ([http://genome.jgi-psf.org/Thaps3/Thaps3.home.html]). The unmasked version of the genome was used in the study. The Thaps3 JGI website also contained the chloroplast and mitochondrial sequences in the file organelle.fasta. Extra sequences that could not be assembled into the genome were available as bottom_drawer.fasta. The GFF formatted filtered gene models were processed to define the locations of the introns, exons, and intergenic regions used in the small RNA analysis. The functional mapping was obtained from a database created and maintained at JCVI. The coordinates for the repetitive regions of the *T. pseudonana* genome were obtained from JGI as a RepeatMasker file.

The one half plate of 454 FLX sequencing generated 305,484 sequences, which were deposited into a fasta formatted file. The slide of ABI SOLiD sequencing generated 17,047,245 sequences, which were sorted by slide region (A1, A2) and barcode (G00032, G31013) and deposited into colorspace formatted files.

#### Initial processing of 454 data

Figure S1A contains a flow chart of the computational analysis steps that were performed for the 454 sequence data. The 5′ and 3′ adapter sequences were matched and removed from the ends of the insert sequences. Sequences that did not possess both adapters (with less than 4 missing nucleotides) were removed from further consideration. To reduce redundancy, the sequences were subjected to clustering using the program CD-hit [36]([http://www.bioinformatics.org/cd-hit/]) at 100% identity for a length similarity of at least 80%.

rRNA is degraded by an RNaseIII mechanism [37], [38], therefore, rRNA degradation products of the selected size are expected to be cloned along with authentic small RNAs. Since the *T. pseudonana* genome is not annotated for non-coding RNA, such as rRNA and tRNA, the consensus sequences were aligned with the non-redundant database “nr” ([http://www.ncbi.nlm.nih.gov/]) and the RNA family database “Rfam” ([39] [http://rfam.sanger.ac.uk/]). Consensus sequences which aligned with these databases with at least 70% alignment and whose best match had functional descriptions of “rRNA”, “tRNA”, or “ribosomal”, were considered to be degraded non-coding RNA and were removed from further consideration. Some degradation products may remain in the candidate pool, since the sequences may have matched “nr” at less than 70% alignment, or the best match to “nr” may not have contained a functional description indicating ribosomal or transfer RNA origin.

The consensus sequences were aligned with the *T. pseudonana* nuclear genome's unmasked chromosomes. 4,287 unique sequences which contained 1 mismatch or less were retained as potential small RNA candidates. The inability of the remaining sequences to align with the *T. pseudonana* assembled genome may be due to sequencing errors in the data set or the assembled genome itself, derivation of the sequence from unsequenced regions of the assembled genome, or mismatches due to different alleles [3]. The percentage of sequences that did not match the assembled genome is similar to the results found in other studies [16], [40]. The sequences were also aligned with the *T. pseudonana* organelle (chloroplast and mitochondria) data, and with the bottom drawer sequence data (a set of sequences that did not assemble with the bulk genomic sequence). At each step, statistics were collected for the counts, lengths, and first nucleotide of each of the sequences.

#### Processing of SOLiD data

Figure S1B contains a flow chart of the computational analysis methodology that was designed and implemented in the current study to process the SOLiD sequence data. The colorspace data was first converted to its basespace equivalent using CLCbio's tofasta software. This program does not reference a genome, so color errors were not detected. Additionally, all SOLiD sequencing reads in the data set were 35 nucleotides, even though the small RNA insert could be any length. Therefore, to remove sequences containing color errors, and to trim off extra nucleotides surrounding the small RNA insert, a methodology was developed in this study using BLAST [35] alignment to the *T. pseudonana* genome. Only sequences which matched the genome perfectly for 100% identity regardless of length, or which had 1-mismatch to the genome, were retained. By retaining the coordinates of the match to extract the sequences from the data file, the sequences could be trimmed to the exact length of the small RNA insert. Sequences that did not match the genome were removed from further consideration. On average, 22% (3,817,895 sequences) of the reads from the individual libraries aligned to the genome, which was higher than when the data was processed with the CLC NGS Cell reference assembly software (7%), or with the ABI Small RNA pipeline (6%). In contrast, in the *T. pseudonana* small RNA library sequenced with the 454 platform, approximately 60% of the reads aligned with the *T. pseudonana* genome. To reduce redundancy, the sequences were subjected to clustering using the program CD-hit [36][http://www.bioinformatics.org/cd-hit/]) at 100% identity for a length similarity of at least 80%. Clustering of the SOLiD data resulted in a set of unique consensus sequences representing an average of 3.9% (675,173 unique sequences) of the total sequences in the original data set. This value fell into a similar range as the 454 sequencing data at 6.5% of the total sequences.

RNA degradation products, such as degraded rRNA and tRNA, were removed from the pool using the locations of these entities. Sequences which aligned with the Rfam database [39] were also removed, except for those sequences that were annotated as miRNA in Rfam. Following removal of RNA degradation products, a pool of 602,087 unique SOLiD sequences (107,711 from sample Tp-EF, and 494,376 from sample Tp-EC) was retained for further processing as potential small RNA candidates. The lengths and first nucleotide of each of the sequences was also tabulated.

A Matlab program was written to indicate strand specificity, localize, and compute coverage of the sequences along each chromosome. A bin-size of 10000 was used, unless otherwise noted. A second Matlab program was written to visualize coverage of the data along the chromosomes as a heatmap, and to accentuate the similarities and differences between the libraries. In both the histograms and heatmaps, the data were normalized by dividing the frequency counts in the bins by the total number of sequences for that particular library.

To determine if the SOLiD small RNA candidate consensus sequences could be further assembled to the genome, each SOLiD data set was processed with CLCbio's CLC Genomics Workbench (version 4.0.3) High-Throughput Sequencing tool for mapping reads to a reference, using the default settings. The assembled sequences were parsed for length and first nucleotide.

#### Prediction of microRNA candidates

Prediction of microRNA candidates was performed using the miRDeep miRNA prediction program [41], with the small RNA candidate consensus sequences in the 18–24 nucleotide size range that aligned with the *T. pseudonana* genome, did not align with repetitive regions of the genome, and did not contain sequence similarity to degraded non-coding RNA. The miRDeep code was modified to allow for the excision of precursors of length +/− 100 nucleotide surrounding the candidate sequence, and to utilize Decypher Timelogic hardware accelerated boards for the alignment. The putative precursor sequences were folded with the RNA folding software *RNAfold* [42] and manually examined to constrain the putative mature miRNA sequence to contain bulges with no more than 3 unpaired nucleotides, and a loop of at least 10 nucleotides in length [43]. The minimum free energy value was required to be less than −20 kcal/mol. All miRNA candidates were required either to be present in at least 2 of the 3 sequence libraries, or the opposite arm of the mature miRNA, known as the miRNA* arm, to be found in at least one of the libraries. MiRNA candidates that passed these tests were further classified as intron, exon, or intergenic, by their location in the predicted gene models.

The miRNA candidate sequences were also aligned with the known mature miRNA sequences in the Sanger microRNA database, miRBase [30]. The query sequences were aligned using the FASTA/ssearch34 alignment program [44]. Software that was written and used previously for a published miRNA search [45] was utilized to examine the results. Matches of 90% identity or better for the first 10 nucleotides of the known miRNAs, were considered “seed” matches [46], and were retained.

#### Prediction of endogenous siRNA candidates

Two types of endogenous siRNA classes were investigated in this study: repeat-associated siRNA and natural antisense transcribed siRNA.

Repeat-associated endogenous siRNA candidates were characterized by aligning the small RNA sequence data with the RepeatMasker repetitive elements in the *T. pseudonana* genome. The matches to each repetitive region family were grouped by transposable element family [47] and tabulated. Additionally, to get an estimate of the sequence similarity of rasiRNA candidates present across the two sequencing platforms, each rasiRNA candidate set was aligned against the other sets using BLAST [35]. Matches that generated an E-value less than or equal to 1×10^−5^ were retained and tabulated.

The small RNA library consensus sequences were analyzed for the presence of endogenous siRNAs, possibly regulating genes via *cis* and *trans* mechanisms. In this study, code was written to determine the orientation of the small RNA transcripts in relation to the genomic DNA and to the coding mRNA. BLAST alignments of the transcripts with the genomic DNA resulted in a reference orientation for the small RNA sequences. The genomic coordinates of the small RNA transcripts were then combined with the gene locations and orientation for the predicted *T. pseudonana* genes in the GFF format file.

#### Prediction of target genes

The prediction of target genes for the predicted miRNA candidate sequences was performed using a few modifications to previously published target prediction software [45]. To predict targets with animal miRNA binding characteristics, the reverse complement matches were classified according to the length of their seed binding, retaining *6-mer* matches with perfect complementary for nucleotides 2–7 of the 5′ end of the small RNA. For plant-like binding, the entire length of the small RNA sequence was examined for similarity, allowing at most 1 mismatch or gap in the 8-nucleotide seed region, and no more than 3 mismatches or gaps in the remainder of the miRNA. The minimum free energy of the pairing was calculated using RNAhybrid [48], and a maximum of −20 kcal/mol was applied to the miRNA:mRNA target hybrids.

The potential target gene data sets used for the target prediction consisted of the *T. pseudonana* filtered gene model transcripts and the *T. pseudonana* EST sequences which had annotated evidence in the GFF format file of a start codon and stop codon. These ESTs represented 3,932 of the 11,890 predicted transcripts in the *T. pseudonana* genome. It was then possible to assign the match locations to occur in the 3′UTR, coding section (CDS), or 5′UTR. The Gene Ontology (GO) terms for the mRNA targets were assigned based on the GO annotation file for the *T. pseudonana* filtered gene models. The GO identifiers were then input into the Gene Ontology (GO) Terms Classification Counter [49]([http://www.animalgenome.org/bioinfo/tools/countgo]), using the EGAD2GO classification filter for higher level grouping.

## Results

### Computational search for RNA interference (RNAi) machinery

The first step in this study required determining if the necessary RNA interference (RNAi) machinery was present in the genome of the diatom, *Thalassiosira pseudonana*, using sequence similarity, pattern similarity, and keyword similarity against the list of known RNAi proteins and motifs (see Text S1). Combining the results of these analyses provided evidence of several key components of small RNA processing machinery, including homologs for one Argonaute protein, two Dicer-like proteins, and three RNA-dependent RNA polymerases. Although *T. pseudonana* did not have full sequence alignments for Argonaute or Dicer, it had good partial alignments. This may be sufficient for the production of small RNAs, since the pattern/motif search via HMM analysis showed that *T. pseudonana* has many of the necessary domains present. *T. pseudonana* does not have evidence of the protein Drosha, which is utilized in miRNA biogenesis in animals [50]. Additionally, *T. pseudonana* does not have the DUF283 domain, so it apparently does not have an animal-like Dicer present. However, this data suggests that diatoms possess one or more of the homologous plant Dicer-like proteins, as well as other important RNAi-related proteins and motifs (Figure S2).

Figure S2 contains a schematic diagram of the key eukaryotic RNAi-related proteins present in the *T. pseudonana* genome. It was found that the matches to the PAZ and PIWI domains fell on the same transcript in the proper order for Argonaute proteins. This finding was confirmed by the subsequent mapping of one Argonaute homolog for *T. pseudonana* on the Superfamily website [51]. There was also evidence of three homologs of RNA dependent RNA polymerase (RdRP), and one protein encoding two Ribonuclease III (RNaseIII) domains (Figure S2). The gene encoding the two RNaseIII domains additionally encodes a PAZ domain, thus showing homology to the *Giardia intestinalis* Dicer gene [52]. A second Dicer-like homolog present in *T. pseudonana* appears to contain the domains for DEAD/DEAH box helicases (DEAD), Helicase conserved C-terminal domain (HELICc), a weak match to the PAZ domain, and a double-stranded RNA binding motif (DSRM) (Figure S2). This is unlike most other organisms with RNAi pathways, which additionally contain two RNaseIII domains in the Dicer protein. Since *T. pseudonana* contains a Dicer-like protein that has two RNaseIII domains and a PAZ domain, these two Dicer-like homologs may act in conjunction. There are examples in *T. brucei* and *Entamoeba histolytica* where Dicer proteins with single RNaseIII domains may act as dimers [52].

A recent study, using different computational analysis techniques, found similar evidence of the RNAi protein machinery present in *T. pseudonana* [53]. In particular, they found evidence of Argonaute, two types of Dicer-like proteins, and several RNA dependent RNA polymerases (RdRp). The main difference between our analyses involves their omission of the PAZ domains in the Dicer-like protein candidates. We found that one putative Dicer-like protein contains a match to the PAZ domain upstream from the two RNaseIII domains. However, since this *T. pseudonana* Dicer-like homolog is missing the DEAD-like helicase, HELIc, and DUF domains, this motif subset is most similar to that of *G. intestinalis*, which also contains only the PAZ and two RNaseIII domains [52].

Homologs for bacterial small RNA processing, such as RNA polymerase sigma factor (rpoS) and the LSM domain of the Hfq chaperone protein [54], [55], were also found in the *T. pseudonana* genome. This suggests that bacterial, as well as eukaryotic, small RNA mechanisms may be possible in *T. pseudonana*, which is consistent with the presence of relatively high numbers of bacterial gene homologs found in the diatom genomes [6]. The presence of bacterial small RNAs was not pursued in this study because the protocol used for 454 sequencing involved a size-selection step, which excluded sequences greater than ∼50 nucleotides in length, and the protocol for SOLiD sequencing involved 35 nucleotide reads. Since bacterial small RNAs are generally 80–100 nucleotides in length, we would not see this type of RNA in our sequence data.

### General characteristics of the small RNA libraries

The small RNA cDNA 454 sequence library was constructed from RNA isolated from exponentially-growing *T. pseudonana* (sample Tp-EF) using established procedures (Methods and [33]). The final gel purification step of the small RNA cDNA library construction revealed the presence of the expected size small RNAs that amplified with the known linkers (Figure 1A). After confirming the presence of cloned small RNAs in a subsample of the library by TOPO cloning and Sanger sequencing, the purified products were sequenced with parallel pyrosequencing on a 454 platform [34], resulting in 305,454 reads.

The two small RNA SOLiD sequence libraries derived from the Tp-EC and Tp-EF cell samples were prepared for sequencing with the ABI SOLiD Small RNA Expression Kit (Life Technologies, Carlsbad, CA). Figure 1B shows an Agilent Bioanalyzer separation of the small RNA library after construction and amplification with the Small RNA Expression Kit. Sequencing generated a total of 17,047,245 sequences in colorspace format.

The length distribution of the small RNA libraries candidate sequences were tabulated after removal of rRNA degradation products and alignment with the *T. pseudonana* genome, and is shown graphically in Figure 1C. For the 454 library, the lengths are consistent with the library construction protocol, as we had size-selected a gel region comprising a range between 20–50 nucleotides. The data show a normal distribution with an average length of approximately 30 nucleotides, with the majority of sequence lengths centered from 28–32 nucleotides. For the two SOLiD libraries, the data do not show a normal distribution, and instead are biased at the extreme ends with major peaks at 18 and 35 nucleotides. Based on the dramatic differences between the 454 and SOLiD data in our study, and previous documentation of bias in SOLiD sequencing of small RNAs [56], we believe that these peaks are not representative of true populations in the small RNA suite of *T. pseudonana*. The 35 nt peak is presumably due to sequences that exceeded the maximum length of the SOLiD sequencing limit. The presence of the 18 nt peak suggests that the actual sequence reads were somehow artificially truncated. Agilent gel analysis (Fig. 1B) does not indicate that the cDNA resulted from substantially degraded RNA. If the 18 nt sequences were artifactual truncations, we reasoned that assembly of them into longer contigs might provide a more accurate representation of the native population. We attempted to assemble the 18 nt data into longer reads using CLC Genomics Workbench. Only about 25% of the 18 nt reads were assembled into longer reads, which had no evidence of clear bias towards truncation at either the 5′ or 3′ end. Thus, the underlying reason(s) for the 18 nt peak are unclear. Based on the established biases in SOLiD data for small RNAs [27], [56], we cannot consider the length distribution data from SOLiD in Fig. 1C as representative of the actual case, whereas the 454 data is. In some other organisms, small RNA lengths are tightly constrained [13]; the 454 results (Fig. 1C) suggests that several types of small RNAs might be present in the diatom. Because the small RNA prediction methods for this study allow for a range of lengths, and use multiple criteria for classification, the truncation of the SOLiD data is not expected to have a significant effect on the subsequent analysis.

The nucleotide present at the 5′ end of a small RNA, which is most frequently a U in most organisms [14], [57], is important due to its link to sorting by the Argonaute protein [58]. Due to truncation of the SOLiD sequences, only the 454 sequences are likely to represent the true 5′ nucleotide [56]. These data showed (Fig. 1D) that G or U was most frequently found at the 5′ end. Since there is only one Argonaute protein annotated in the *T. pseudonana* genome, which must process the different types and lengths of small RNAs, it may be adapted to work with either a 5′ U or G. Another possibility is that other currently underdetermined mechanisms for cleavage and processing are present in *T. pseudonana*.

#### Comparative mapping along the chromosomes

**Figure 2:**
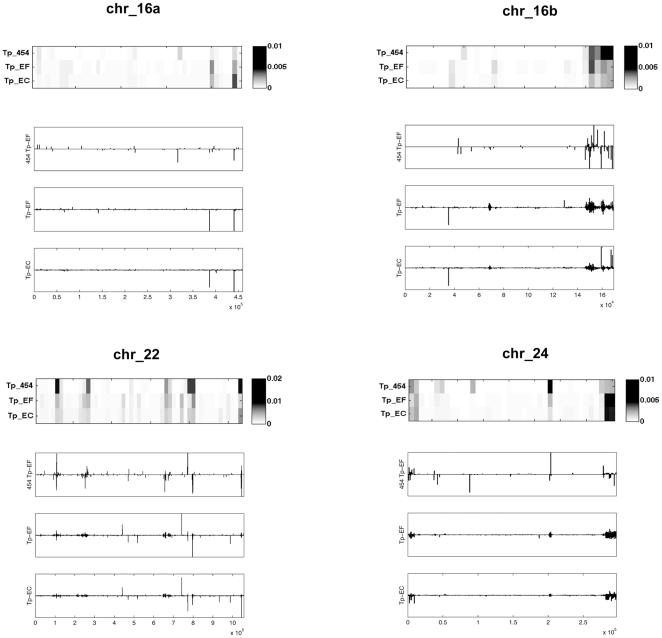
Heatmaps and histograms of small RNA candidate abundance mapped along chromosomes 16a, 16b, 22, 24. All alignments to the *T. pseudonana* genome were binned, normalized, and then plotted along the length of the chromosome as a heatmap and as a histogram. The intensity of the spot on a heatmap denotes the abundance of sequences generated at the particular site relative to the total dataset for that sample, with darker colors depicting higher abundance. Additionally, the alignment coordinates of the sequences were binned into histograms along each chromosome. Values above the x-axis signify that the small RNA was transcribed along the plus strand of DNA, and below the x-axis for the minus strand. Each row of the heatmap represents a different sample library in the following order: Tp_454data, Tp_EF, Tp_EC. Bars above the line represent the plus strand and bars below the line represent the complimentary strand. A binsize of 10000 was used for both types of plots.

To compare the expression profile between the small RNA libraries, each set of sequences was binned, normalized, and represented in a heatmap plot along the length of the chromosomes (Figure 2). As an example, Figure 2 shows the location and frequency of generation of small RNAs along chromosomes 16a, 16b, 22, and 24. (Figure S3 contains the mappings for all of the chromosomes.) The small RNAs do not appear evenly distributed on the chromosomes, but instead are grouped into clusters or hotspots. These results are consistent with other studies of small RNAs [59]. The similarity between the 454 data and the SOLiD data, indicates that the truncated SOLiD sequences are still useful for analysis, and more importantly, gives strong evidence that there exists a core complement of small RNAs expressed in *T. pseudonana*. Sequences uniquely present in the SOLiD data (Fig. 2) are consistent with the greater depth of coverage using this approach.

### Prediction of miRNA candidates

Our initial small RNA characterization analysis focused on searching for miRNA candidates. The sequences for all three libraries were pooled and input into the miRNA prediction program, miRDeep [41], which predicts miRNAs according to statistical evidence of Dicer processing. In the 18–24 nucleotide size range of the small RNA sequences, the structures of possible precursor sequences obtained from flanking regions of genomic DNA produced by the folding software *RNAfold* were examined for quality of intramolecular hairpin candidates. In particular, the hairpins were considered good candidates if they contained bulges of less than 3 unpaired nucleotides in the mature miRNA stem region, and a loop length greater than or equal to 10 nucleotides. The base pairing requirement is based upon the accepted criteria for annotating microRNA [43]. The loop length parameter is based on statistics gathered from known miRNAs in the Sanger miRBase from a previous study [45], and by experimental evidence that Drosha RNase III processing of animal miRNAs requires a loop length greater than 10 nucleotides [60]. Additionally, the minimum free energy value, as calculated by *RNAfold*, for the precursor hairpin was required to be less than −20 kcal/mol. For the sequences that met the basic folding criteria for base pairing, loop length, and minimum free energy value, two subsequent steps were taken before classifying the sequence as a miRNA candidate. First, to strengthen the validity of the mature miRNA expression, the mature miRNA sequence had to be either present in the same polarity in at least 2 of the 3 sequence libraries, or the opposite arm of the mature miRNA, known as the miRNA* arm, had to be present in at least one of the sequence libraries. The occurrences of both the miRNA and miRNA* arms strongly suggests the ability to form miRNA∶miRNA* duplexes during miRNA biogenesis [14]. Second, to rule out misannotation of repeat-associated siRNA as miRNA candidates, the sequence was not permitted to align with a repetitive region in the genome.

Of the structures that met these criteria, 29 miRNA candidates were predicted over the 18–24 nucleotide size range that is characteristic for miRNAs. These miRNA candidates originated from reads present in the SOLiD libraries, rather than the 454 library, presumably due to the lack of sequencing depth with the 454 platform coupled with our stringent criteria for miRNA prediction. The percent of miRNAs relative to the total number of genes in the genome is lower than described in other organisms (0.25% relative to 0.5–1.5%) [11]. Table S1 contains a list of the miRNA candidates with information about their length, number of mature miRNA reads, number of miRNA* reads, genome coordinates, terminal loop length, precursor length, and the sequences that contributed to their predition. Figure S4 contains the secondary structures for the predicted miRNAs.

The precursor sequences of the miRNA candidate hairpin structures ranged in length from 70 to 132 nucleotides, with an average length of 107 nucleotides and a median length of 110 nucleotides. These lengths are within the range of plant miRNA precursors, which range from 50 to more than 350 nucleotides [61]. Longer lengths may have been precluded due to the limits in the miRDeep prediction algorithm. These results are different from the miRNAs present in *Chlamydomonas*, for which the majority of the miRNA precursors ranged from 150 to 729 nucleotides [15].

The predicted miRNA candidates were also mapped relative to the *T. pseudonana* annotated genes. Intergenic regions produced 24% of the miRNA candidates, while approximately 60% were found in exons, and only 6% in introns. These results suggest similarity to the characteristics of plant miRNAs, for which a greater proportion of the candidates occur within intergenic, rather than intronic, regions of the genome [62]. These results are also similar to those found in a small RNA study of the moss, *Physcomitrella patens*, which reported 43% of the miRNAs were generated from intergenic regions, and the remainder of miRNA loci originated from exons, introns, and exon-intron boundaries [63]. In the green algae, *Chlamydomonas*, the majority of miRNAs were generated from intronic, rather than intergenic regions as they are in plants [16]. In animals, the majority of miRNA are transcribed from intronic regions [64]. The lower number of miRNA candidates originating from introns in the *T. pseudonana* data set relative to some other organisms may be due to the small size and amount of intronic sequence in the *T. pseudonana* genome. It is estimated that in the *T. pseudonana* genome, there are 1.52 introns/gene, with an average size of 125–135 bp, and a median length of 90–92 bp [6]. Additionally, it is estimated that 39% of the genes are single-exon gene models.

To determine if known conserved miRNAs were present, the 29 putative *T. pseudonana* miRNA candidate sequences were compared to the Sanger miRBase. The seed region was examined for homology to known miRNAs. When enforcing a 90% identity constraint for the first 10 nucleotides, only 9 sequences matched the Sanger miRBase. On visual inspection, the hairpins did not show significant structural homology to the corresponding miRNA in miRBase. The lack of strong phylogenetic conservation of our small RNA candidates to those found in other organisms was not surprising. Recently, it was determined that although the unicellular green algae *Chlamydomonas reinhardtii* possess miRNA genes, they do not share miRNA sequence conservation with plants and animals [15], [16]. Since the diatom is a unicellular brown algae, which is not represented in the Sanger miRBase, it is very likely that diatom miRNAs will provide a new set of miRNAs to the database.

### Prediction of endogenous siRNA candidates

Short interfering small RNAs (siRNAs) are generated from double stranded RNA in the transcriptome by several mechanisms [65]. The two types of siRNAs that were explored in this study were repeat-associated siRNAs and natural antisense transcribed siRNAs.

Repeat-associated siRNAs, or rasiRNAs, have been found to silence homologous retrotransposons and other repetitive sequences in the genome of an organism in sense and antisense orientations. In this study, the small RNA sequence data was aligned with the RepeatMasker repetitive elements in the *T. pseudonana* genome. A total of 857 unique small RNA sequences from the 454 small RNA candidate pool mapped to the repetitive elements (15.9% of the small RNA candidate pool), while the counts were 8093 and 19,006 for the SOLiD Tp-EF (7.1%) and SOLiD Tp-EC (3.7%) libraries, respectively. These percentages are notable, considering that only approximately 2% of the *T. pseudonana* genome is composed of interspersed repeats and transposable elements [3], [6]. In contrast, the *Chlamydomonas* small RNA study [16] found a lower percentage of repeat-associated small RNAs in their library than in the genome. The amoeba *Dictyostelium discoideum*, on the other hand, was found to have approximately 68% of small RNAs derived from the DIRS-1 retrotransposon [40]. DIRS-1 is the most abundant retrotransposon in the amoeba, and is believed to function as centromeres at mitosis. Small RNAs found in repetitive regions near centromeres are also a hallmark of gene regulation in yeast, *S. pombe* [66]. Currently, no evidence for centromeric sequences has been found, based on G+C content, transposable element distribution, or gene poor regions in the *T. pseudonana* genome [6]. Therefore, no statement can be made concerning the relation of the locations of the repetitive regions or the repeat-associated small RNAs to the centromeres. However, the abundance of small RNA sequences derived from the repetitive regions of the *T. pseudonana* genome suggests that they play important roles in regulation and silencing of the transposable elements. The transcriptional activity of transposable elements in diatoms is known to be elevated under stress conditions, such as nitrogen starvation [67].

**Figure 3:**
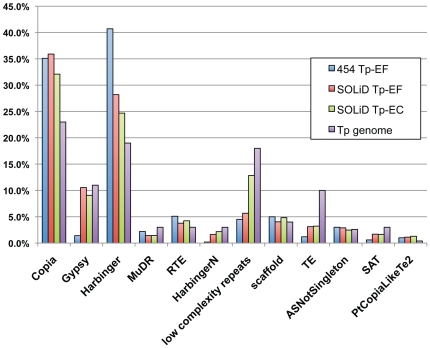
Percentage of small RNA sequences in each repetitive element class in the *T. pseudonana* genome for each library.

Figure 3 contains the results of the repeat-associated siRNA mapping with the types of repetitive elements found in the *T. pseudonana* genome [47]. For both of the SOLiD libraries, the long-terminal repeat (LTR) retrotransposon class, which contains Copia and Gypsy, was expressed in the highest abundance. The DNA transposons, Harbinger and MuDR, were expressed in lower abundance. This is to be expected, since the LTR retrotransposons comprise a larger proportion of the *T. pseudonana* genome [3], [6], [67]. This result is different from the 454 data, where the DNA transposons were expressed at a higher percentage than the LTR retrotransposons. This may be due to a bias in the processing or in the lower quantity of sequences in the 454 data set.

By aligning the rasiRNA candidates against each library using BLAST [35], a high percentage of similarity between the two technologies was found. In particular, for the 454 sequence data: 66% of the rasiRNA candidates contained matches to the SOLiD TP-EF rasiRNAs, 79% contained matches to the SOLID-EC rasiRNAs, and 82% contained matches to the combined SOLiD rasiRNA candidate pool. Between the SOLiD libraries, 80% of the SOLiD Tp-EF rasiRNA candidates contained matches to the SOLiD Tp-EC rasiRNAs. The detection of these candidates across the libraries suggests the expression of a core set of rasiRNAs.

**Figure 4:**
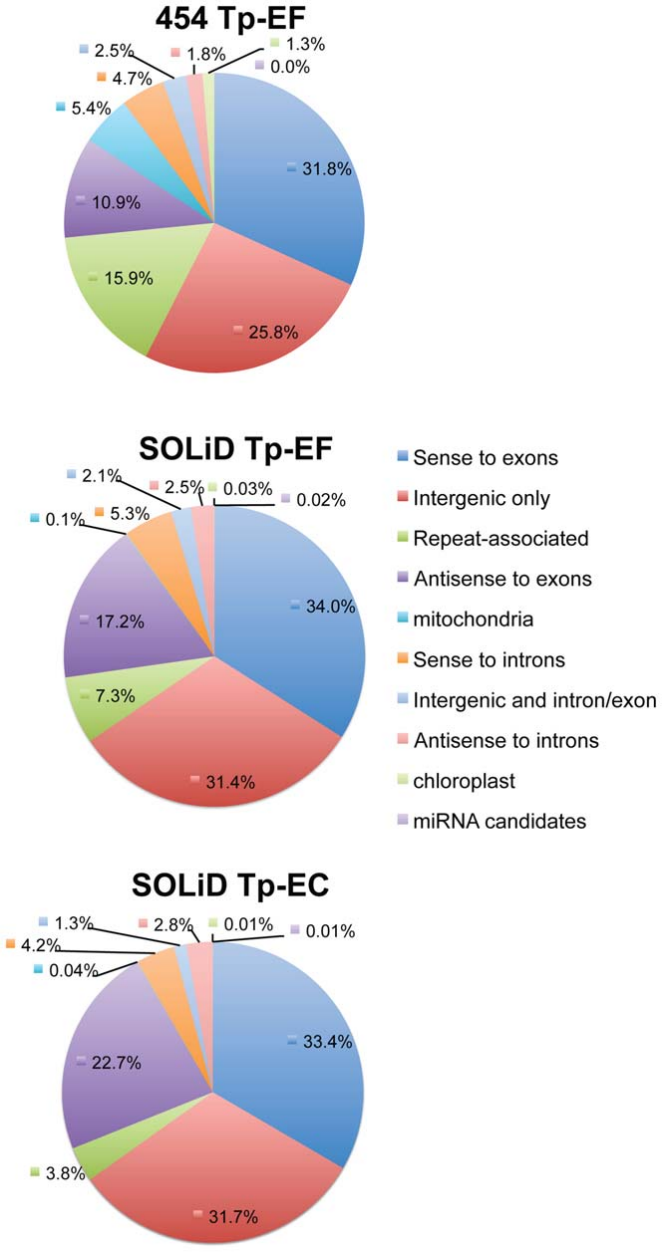
Summary of the small RNA sequence distribution in the *T. pseudonana* genome for each library.

Natural antisense transcript-derived siRNA (nat-siRNA) are produced from double stranded RNA formed from by transcription of overlapping gene regions. These endogenous short interfering RNAs may act as cis or trans regulatory elements. From the pool of *T. pseudonana* small RNA sequences, after removing repeat-associated and miRNA candidates, the sequences were examined for their orientation in relation to the genomic DNA and the predicted gene transcripts. As shown in Figure 4, the majority of the endogenous siRNA candidates were transcribed from sense, antisense, or intergenic RNA, suggesting a possible natural antisense regulatory role in the cell. Antisense transcription is prevalent in the mammalian genome, with original estimates that 20% of the human transcripts may form sense-antisense pairs [68], to later estimates of twice that value [69]. Regulation by antisense transcription has also been reported in the plant, *Arabidopsis thaliana* [70]. The *Chlamydomonas reinhardtii* small RNA study [16] classified over half of their total small RNA reads as originating in protein-coding genes and intergenic regions. Therefore, the abundance of putative natural antisense transcript-derived siRNAs found in the *T. pseudonana* small RNA candidate pool is in line with these other studies.

The endogenous sense-antisense siRNA candidates analysis determined that there were a large number of sequences transcribed from intergenic regions, which is consistent with studies of plant small RNAs [71]. However, the most interesting candidates were transcribed in the antisense direction to the introns and exons, or mapped to both intergenic and protein coding regions. These characteristics suggest that the small RNAs could form double-stranded RNA with the protein coding genes, generating endogenous siRNAs that have regulatory properties. Some of the antisense transcription candidates were highly represented in the data set.

### Prediction of mRNA targets for the putative miRNAs

Target prediction was performed for all of the putative miRNAs characterized in this study. Using binding characteristics of both plant and animal targets, a substantial list of possible targets was created. The percentage of predicted targets for the putative miRNAs relative to the total number of ESTs searched in *T. pseudonana* using the plant criteria was 5%, and using the animal criteria was 18%. For both plant and animal criterion, c.a. 96% of the predicted binding sites in targets were within the coding section (CDS), 3% were within the 3′-UTR, and 1% were in the 5′UTR. A majority of the targets binding in the CDS is a characteristic of plant miRNA target binding [72]. Table S2 contains the predicted targets for the miRNA candidates.

The target genes were also grouped according to their Gene Ontology (GO) terms. The majority of target gene functions are involved in metabolism, membrane transport, and nucleic acid metabolism. However, these gene functions also comprise the majority of gene functions in the entire set of annotated *T. pseudonana* filtered gene models. Therefore, in an attempt to further tease out functional bias, the ratio of the fraction of the target gene functional representation to the overall gene set was compared. Table S3 contains the list of the top gene functions, in descending ratio of occurrence, for the targets that were predicted for the miRNA candidates. The predicted target genes had a high proportion of functions related to post-translational modification, in addition to metabolic processing genes. Because of the stringency of the target matches, and in some cases, the presence of multiple binding sites, many of the target genes demonstrate potential to control key cellular processes.

The differences observed between the SOLiD and 454 sequence data sets are expected due to potential biases in the experimental and sequencing approaches [27]. In the sample preparation prior to 454 sequencing, the total RNA was treated with a PEG/NaCl precipitation step followed by PAGE size-selection to reduce the rRNA degradation product content [33]. In the ABI SOLiD protocol, on the other hand, the total RNA was eluted through a flashPAGE device to achieve this same result. The two protocols also used different ligation adapters to selectively bind to the small RNAs. The sequencing technologies are vastly different, whereby 454 involves pyrosequencing in picotiter plates [34], and SOLiD involves sequencing-by-ligation on a support [24]. The data produced by these two different sequencing platforms also requires different processing and analysis procedures. In 454 sequencing, the bases are determined individually, while in SOLiD sequencing, it is necessary for the base-calling procedure to interpret one color as a 2-base pair. This SOLiD colorspace data is converted to its equivalent base space format, and then mapped to a reference genome before further analysis can be performed. Finally, the quantity of data produced by 454 sequencing is approximately 100-fold less than with SOLiD. The most recent estimates claim that a typical 454 sequencing run generates 600 Mb of data ([http://www.454.com/]), while SOLiD generates over 60 Gb of sequence per run ([http://www3.appliedbiosystems.com/]). Although these differences in techniques can be used to explain disparities between the 454 and SOLiD data, these biases also serve to strengthen the validity of general trends exhibited across both sets.

Computationally, the abundance of data produced from the SOLiD sequencing has both advantages and disadvantages. The massive data set is beneficial because the increase in data accentuates the trends in the data. The disadvantages of the large sequence data set include lengthier computational processing times and the requirement of more storage space. We identified biases in the average sequence length of the small RNAs, similar to a previous study [56]. Additionally, in our study, we found that approximately 80% of the data did not align with the genome and had to be discarded. Low alignment rates, ranging from 50–80% unmapped reads, have been reported for SOLiD sequencing, due to intolerance of the dibasic color reads to mismatches and polymorphisms in the genome [73], [74].

The methodology presented in this study provides the steps necessary to discover small RNA genes in next generation sequence data, and to perform a comparative analysis of different libraries of sequence data. This is especially important in organisms which lack hyper-abundant small RNAs. In control experiments, we were unable to detect several of the more highly represented small RNAs in the datasets using Northern analysis, confirming their low abundance. Unlike the ABI SOLiD Small RNA data analysis pipeline, the method described here contains no filtering of the data by Sanger miRBase [30], thereby freeing the analysis to pursue all types of small RNAs. By using the BLAST program [35] to align the reads to the genome, all length classes are represented and all locations of the matches are collected, while trimming the adapters from the ends of the reads. Additionally, this method assembled, on average, two or three times more reads to the genome than the ABI SOLiD Small RNA pipeline and CLCbio's NGS Cell program, thereby producing a large data set for further analysis. Although the quality of SOLiD-based analyses on small RNAs has been substantiated by comparison with other methods [27], [56], these studies have also documented biases comparing the different methods. One bias is introduced during sample preparation; comparison of the “Modban” or “SREK” sample preparation methods showed substantial differences in the representation of specific small RNAs in sequenced libraries [27]. Another bias occurs in the actual sequencing method; a comparison of SOLiD vs. two Illumina sequencing methods indicated a more dispersed distribution of length and higher frequency variation for nucleotides near the 3′- and 5′-ends when using SOLiD, and in one dataset, the SOLiD approach indicated a smaller size class (in this case 20 nt) of small RNAs than two Illumina methods [27]. As long as these biases are considered, SOLiD (which has a greater depth of coverage) has proven to be a valid approach to characterizing small RNAs [26], [27], [28], [29].

## Discussion

Combining the results from all of the sequencing data analyses, this study demonstrates strong evidence of small RNA expression in the transcriptome of the diatom, *Thalassiosira pseudonana*. Figure 4 shows the percentage of small RNA sequences that may be acting as endogenous siRNAs, repeat-associated RNAs, or miRNAs. The similarities between the expression profiles of the *T. pseudonana* small RNA libraries confirm that the common small RNAs discovered in this study are valid, since they were generated from multiple cell samples, as biological replicates, and with two different protocols and sequencing approaches, as technical replicates. The agreement between the libraries is most clearly visible as hotspots of transcription for the small RNAs in the histogram and heatmap distribution plots along the chromosomes (Figure 2; Figure S3). An overall similarity between the libraries for the predicted repeat-associated siRNAs (Figure 3) and over the entire small RNA profile (Figure 4), can also be observed. These features demonstrate the existence of a core group of small RNAs that is expressed in the diatom.

Several pieces of evidence suggest that diatom small RNAs are more similar to plant than animal small RNAs. The *T. pseudonana* genome lacks the protein Drosha, which is involved in animal miRNA biogenesis. It also lacks a DUF283 domain, which is characteristic of animal Dicer proteins. Other plant-like characteristics include 1) the predominance of siRNAs in the small RNA candidate pool [75], 2) similar lengths of miRNA precursors, and 3) most predicted targets in the coding regions.

The presence of small RNA in *T. pseudonana* unlocks the potential to discover small RNA gene regulation mechanisms and gene regulatory networks in the diatom. Understanding the gene regulation mechanisms involved in carbon fixation, nitrogen, iron, and silicon utilization in the diatom creates the possibility to manipulate these processes for beneficial environmental purposes and technological advances. Furthermore, because of the unique evolutionary footprint of diatoms and other chlorophyll c algae, which has been greatly influenced by eukaryotic-eukaryotic endosymbioses, the small RNA repertoire found in diatoms may provide insight into the evolutionary history of regulatory small RNAs.

### Accession numbers

Sequencing data is available at the NCBI Sequence Read Archive (SRA) ([http://www.ncbi.nlm.nih.gov/Traces/sra/]). Accession number SRA027169 contains the Tp-EF 454 sequence data. Accession number SRA027146 contains the Tp-EF G00032 SOLiD sequence data. Accession number SRA027168 contains the Tp-EC G31013 SOLiD sequence data.

## Supporting Information

Figure S1 **Flow charts of the computational analysis steps performed on the** ***T. pseudonana*** **data sequences. S1A - Flow chart for the 454 data sequences. S1B - Flow chart for the SOLiD data sequences.**
(PDF)

Figure S2 **Evidence of RNAi machinery in the** ***T. pseudonana*** **genome.** Schematic diagram of the *T. pseudonana* genes demonstrating homology to the Argonaute, Dicer, and RNA dependent RNA polymerase (RdRp) families of proteins. The gene names refer to the filtered gene models from the *T. pseudonana* JGI website (http://genome.jgi-psf.org/Thaps3/Thaps3.home.html). In parentheses below each motif are the residue coordinates and HMM E-value for the motif in the gene. The typical Dicer motifs, DUF283 and RNaseIII, which were not find in transcript 20605 are denoted with an ‘X’ through the motif. Abbreviations used in this diagram: DEAD - DEAD-like helicase, DSRM - Double-stranded RNA binding domain, DUF - DUF283 domain, Hel-C - Helicase C-terminal domain, PAZ - PAZ domain, PIWI - PIWI domain, RdRp - RNA dependent RNA polymerase, RNaseIII - Ribonuclease III domain.
(PDF)

Figure S3 **Heatmaps and histograms of small RNA candidate abundance mapped along all of the** ***T. pseudonana*** **chromosomes.**
(PDF)

Figure S4 **Predicted secondary structures of the microRNA candidates in the** ***T. pseudonana*** **small RNA libraries.** The precursor sequences have been folded with RNAfold. The mature miRNA portions are highlighted in yellow. The miRNA* arm, if present, is highlighted in blue.
(PDF)

Table S1 **List of microRNA candidates in the** ***T. pseudonana*** **genome.** This file contains the information about the sequences that folded into hairpin structures, and were thereby classified as miRNA candidates. The information includes the length, number of reads, mature miRNA sequence, terminal loop length, location of match to T. pseudonana genome (chr∶start-end), orientation, mature miRNA structure in RNAfold notation, minimum free energy of the hairpin structure, miRNA* name and sequence if present, precursor length, and sequences that contributed to the prediction.
(XLS)

Table S2 **List of the predicted targets for the miRNA candidates.** The target prediction uses plant-like and animal-like target binding characteristics.
(XLS)

Table S3 **Predicted target gene functional categories for miRNA candidates.** Functional categories are in an ordered list as a ratio to the frequency of the functional groups in the genome.
(XLS)

Text S1 **List of proteins and motifs used in search for RNAi machinery in** ***T. pseudonana*** **genome.**
(DOC)
