# Supplementary material for: Characterization of the Small RNA Transcriptome of the Diatom, Thalassiosira pseudonana
Source: PLoS One. 2011 Aug 12;6(8):e22870. doi: 10.1371/journal.pone.0022870 (PMC3155517; doi:10.1371/journal.pone.0022870)
Supplement: Figure S1 — Flow charts of the computational analysis steps performed on the T. pseudonana data sequences. S1A - Flow chart for the 454 data sequences. S1B - Flow chart for the SOLiD data sequences. (PDF) [file pone.0022870.s001.pdf]

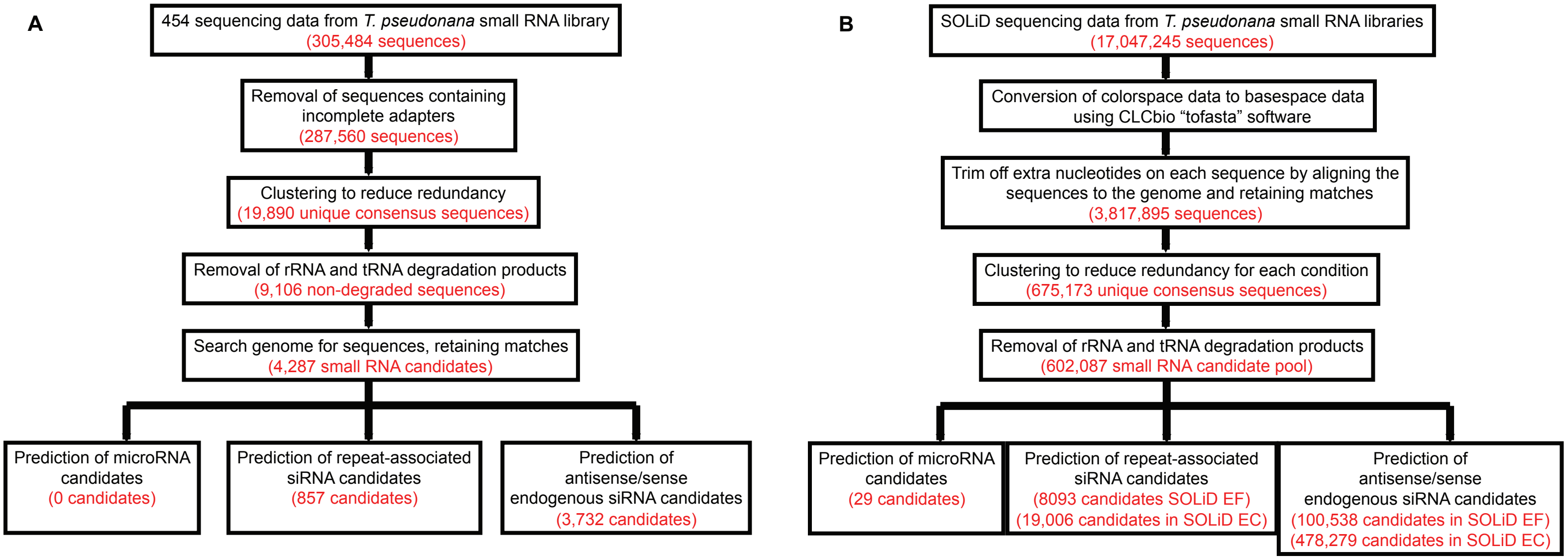
A
454 sequencing data from T. pseudonana small RNA library
(305,484 sequences)
Removal of sequences containing incomplete adapters
(287,560 sequences)
Clustering to reduce redundancy
(19,890 unique consensus sequences)
Removal of rRNA and tRNA degradation products
(9,106 non-degraded sequences)
Search genome for sequences, retaining matches
(4,287 small RNA candidates)
Prediction of microRNA candidates
(0 candidates)
Prediction of repeat-associated siRNA candidates
(857 candidates)
Prediction of antisense/sense endogenous siRNA candidates
(3,732 candidates)
B
SOLiD sequencing data from T. pseudonana small RNA libraries
(17,047,245 sequences)
Conversion of colorspace data to basespace data using CLCbio “tofasta” software
Trim off extra nucleotides on each sequence by aligning the sequences to the genome and retaining matches
(3,817,895 sequences)
Clustering to reduce redundancy for each condition
(675,173 unique consensus sequences)
Removal of rRNA and tRNA degradation products
(602,087 small RNA candidate pool)
Prediction of microRNA candidates
(29 candidates)
Prediction of repeat-associated siRNA candidates
(8093 candidates SOLiD EF)
(19,006 candidates in SOLiD EC)
Prediction of antisense/sense endogenous siRNA candidates
(100,538 candidates in SOLiD EF)
(478,279 candidates in SOLiD EC)
