# Supplementary material for: Characterization of the Small RNA Transcriptome of the Diatom, Thalassiosira pseudonana
Source: PLoS One. 2011 Aug 12;6(8):e22870. doi: 10.1371/journal.pone.0022870 (PMC3155517; doi:10.1371/journal.pone.0022870)
Supplement: Figure S2 — Evidence of RNAi machinery in the T. pseudonana genome. Schematic diagram of the T. pseudonana genes demonstrating homology to the Argonaute, Dicer, and RNA dependent RNA polymerase (RdRp) families of proteins. The gene names refer to the filtered gene models from the T. pseudonana JGI website (http://genome.jgi-psf.org/Thaps3/Thaps3.home.html). In parentheses below each motif are the residue coordinates and HMM E-value for the motif in the gene. The typical Dicer motifs, DUF283 and RNaseIII, which were not find in transcript 20605 are denoted with an ‘X’ through the motif. Abbreviations used in this diagram: DEAD - DEAD-like helicase, DSRM - Double-stranded RNA binding domain, DUF - DUF283 domain, Hel-C - Helicase C-terminal domain, PAZ - PAZ domain, PIWI - PIWI domain, RdRp - RNA dependent RNA polymerase, RNaseIII - Ribonuclease III domain. (PDF) [file pone.0022870.s002.pdf]

Argonaute

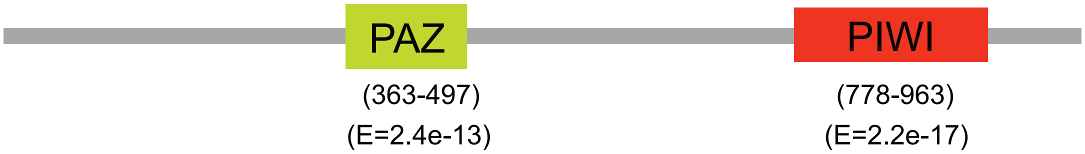

Jgi|Thaps3|20630|estExt_fgenesh1_pg.C_chr_10162

Dicer-like proteins

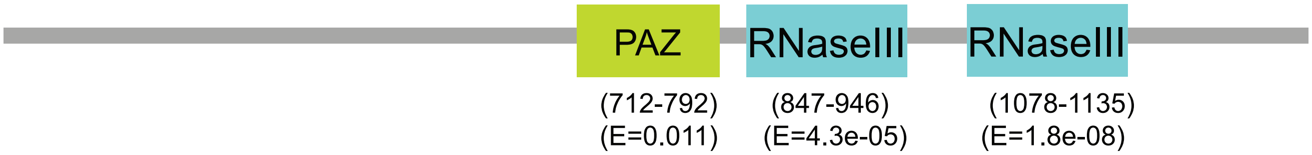

Jgi|Thaps3|264746|thaps1_ua_kg.chr_20000087

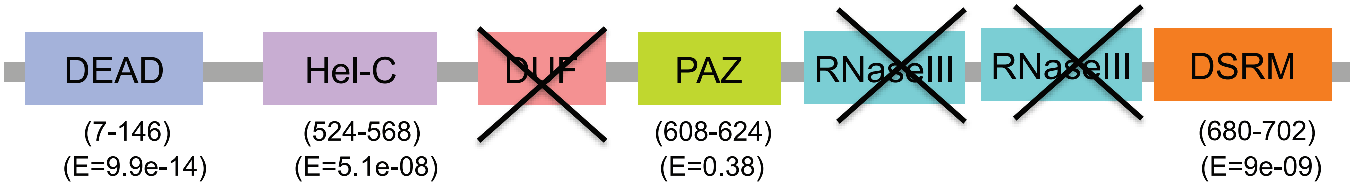

Jgi|Thaps3|20605|estExt_fgenesh1_pg.C_chr_10107

RNA dependent RNA polymerase (RdRP)

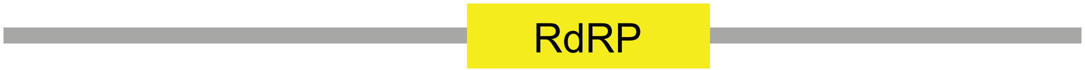

Jgi|Thaps3|5028|fgenesh1_pg.C_chr_5000070 (nt #730-763) (E=4.4e-06)

Jgi|Thaps3|9018|fgenesh1_pg.C_chr_12000099 (nt #591-610) (E=0.00014)

Jgi|Thaps3|22277|estExt_fgenesh1_pg.C_chr_40436 (nt #1081-1129) (E=0.00018)
