# Supplementary material for: Characterization of the Small RNA Transcriptome of the Diatom, Thalassiosira pseudonana
Source: PLoS One. 2011 Aug 12;6(8):e22870. doi: 10.1371/journal.pone.0022870 (PMC3155517; doi:10.1371/journal.pone.0022870)

chr_1

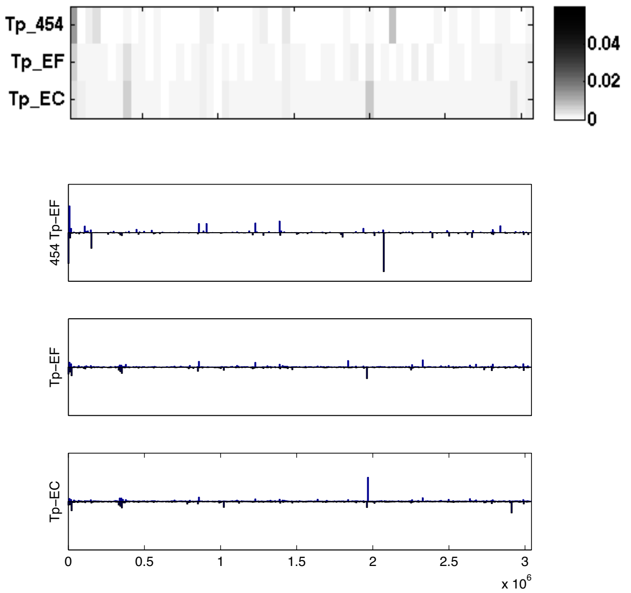

chr_2

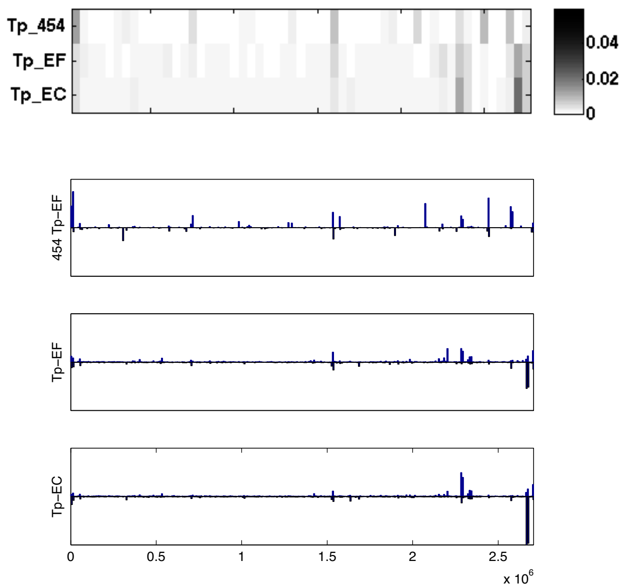

chr_3

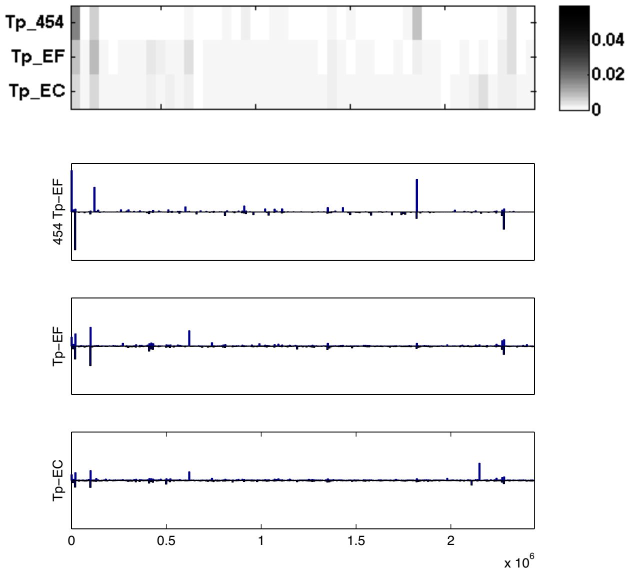

chr_4

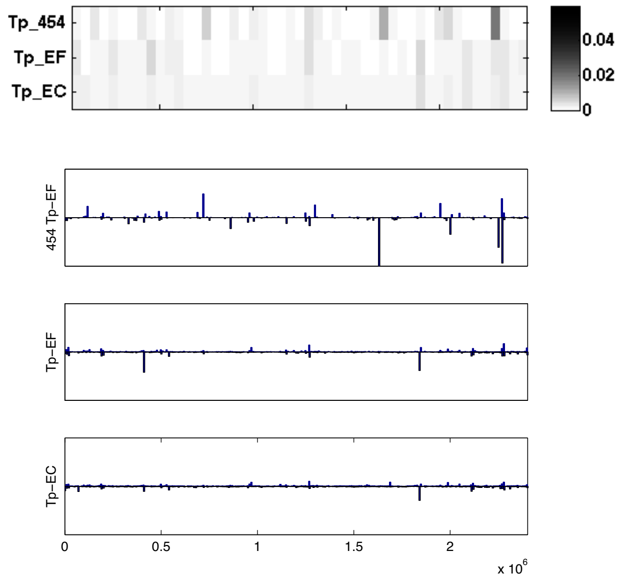

chr_5

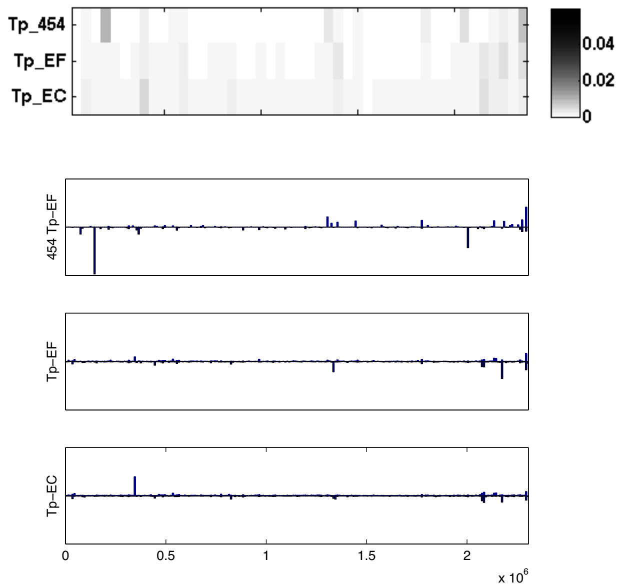

chr_6

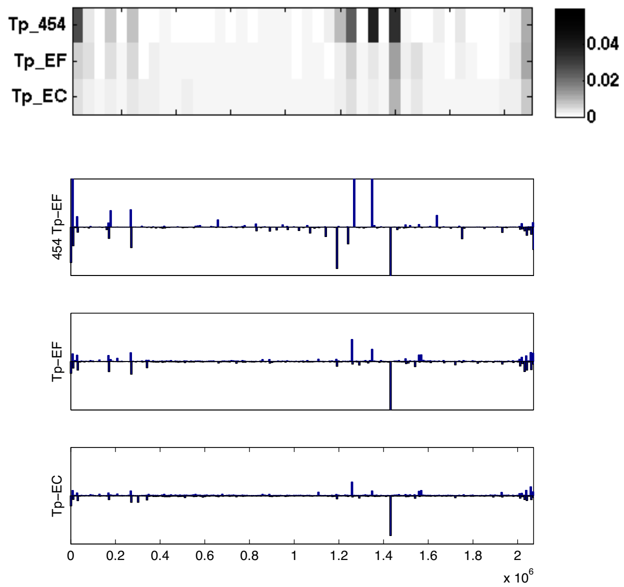

chr_7

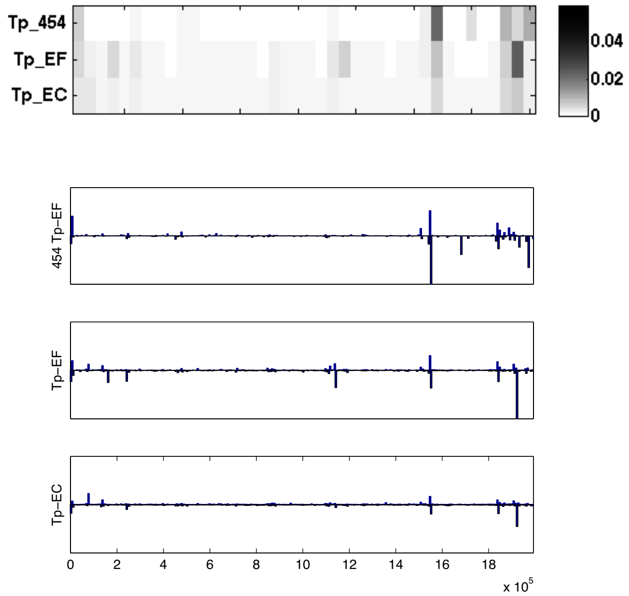

chr_8

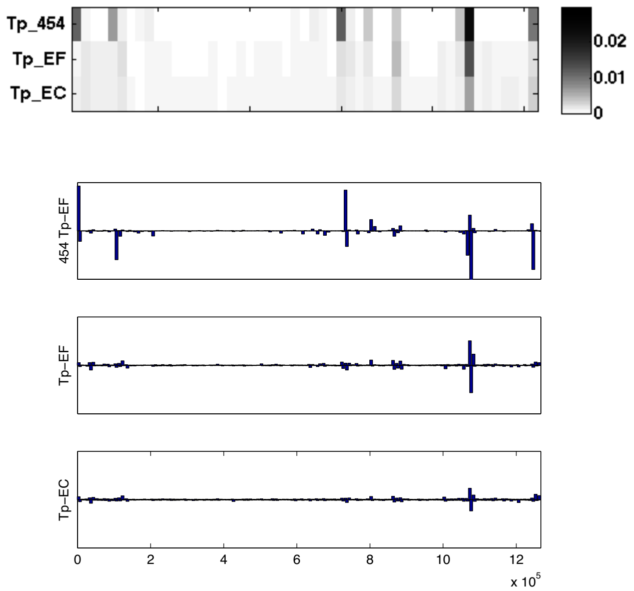

chr_9

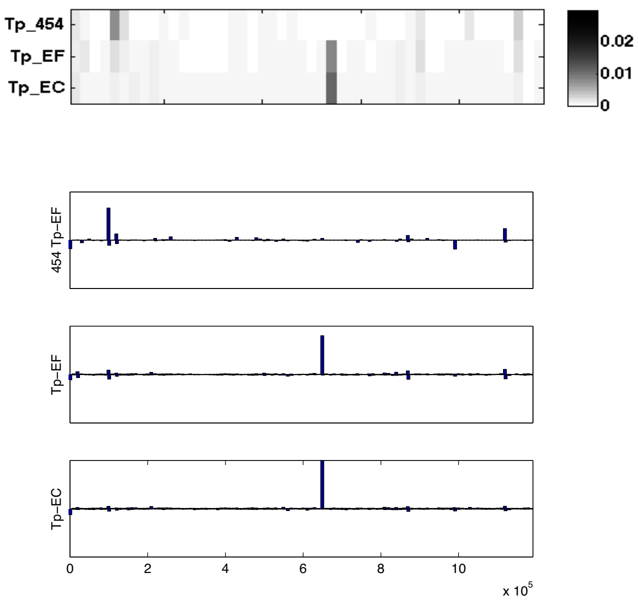

chr_10

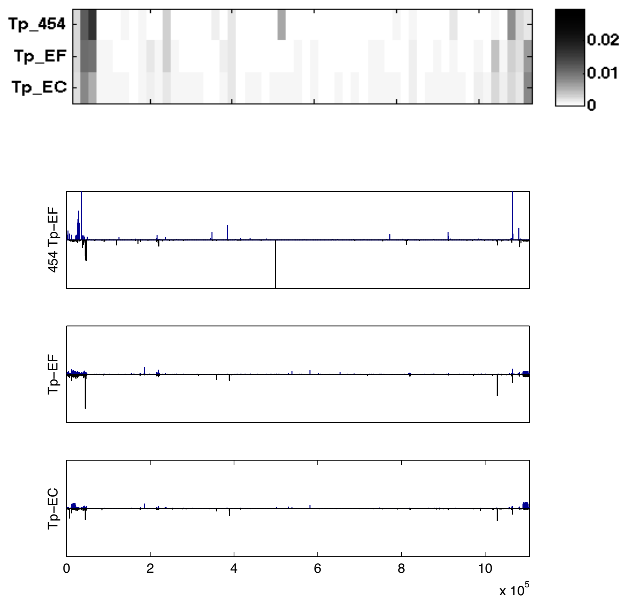

chr_11a

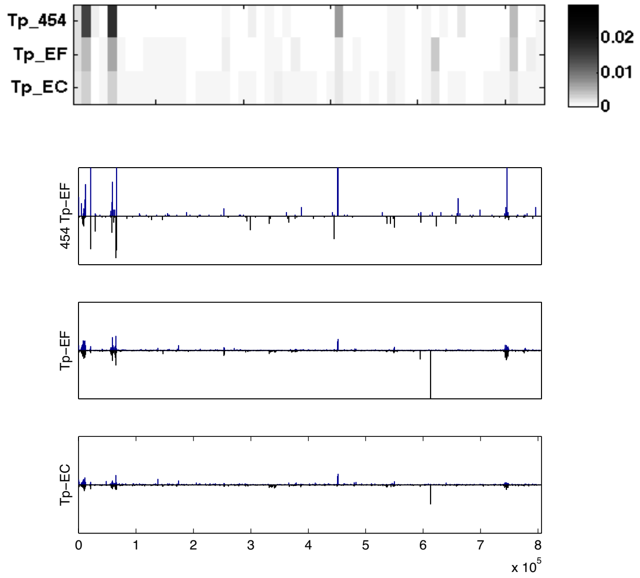

chr_11b

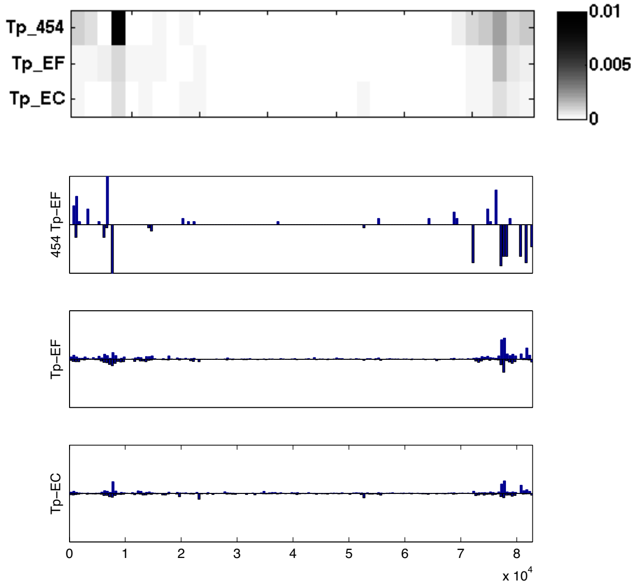

chr_12

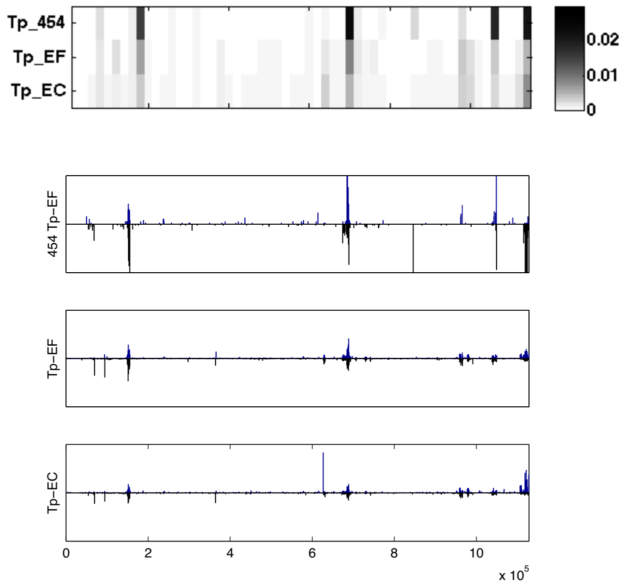

chr_13

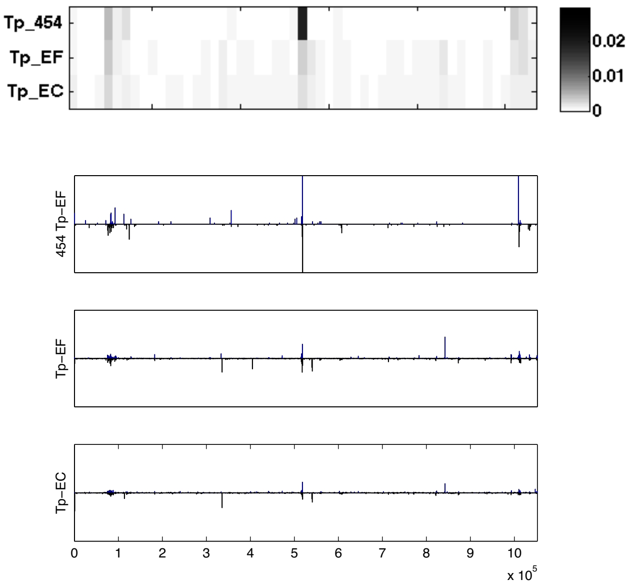

chr_14

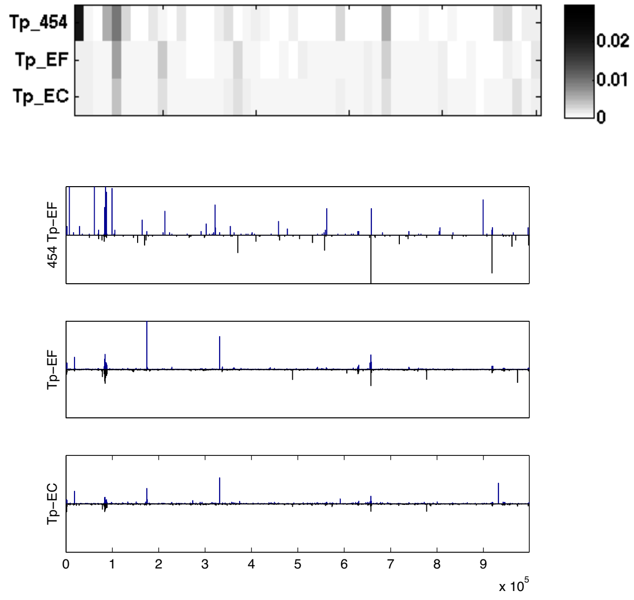

chr_15

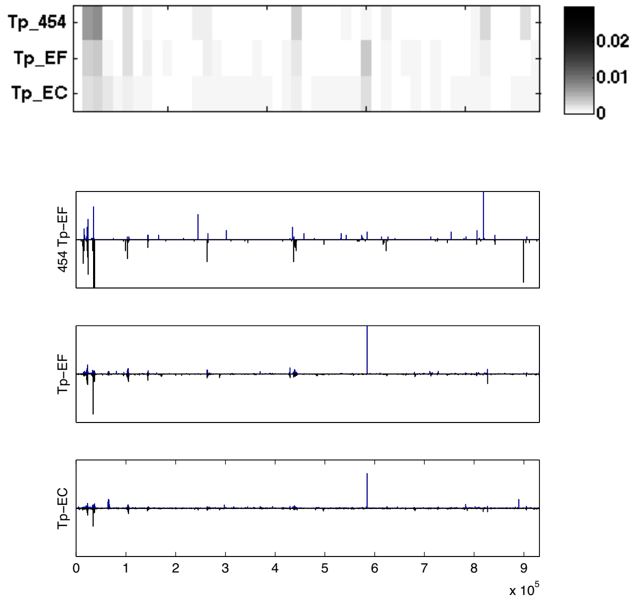

chr_16a

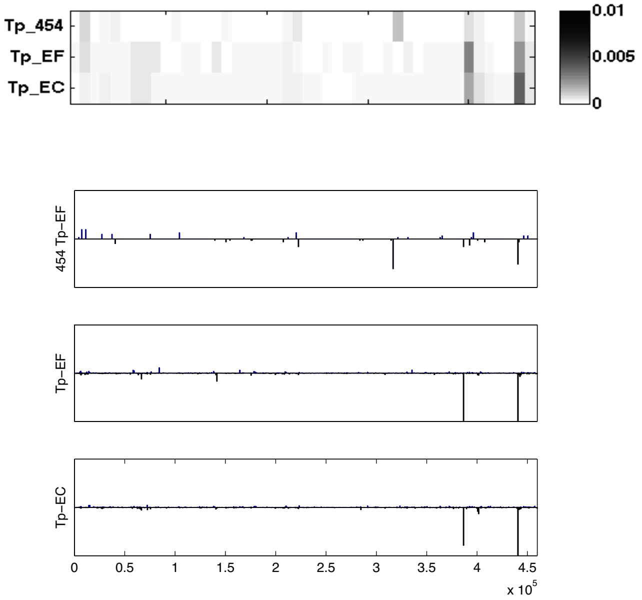

chr_16b

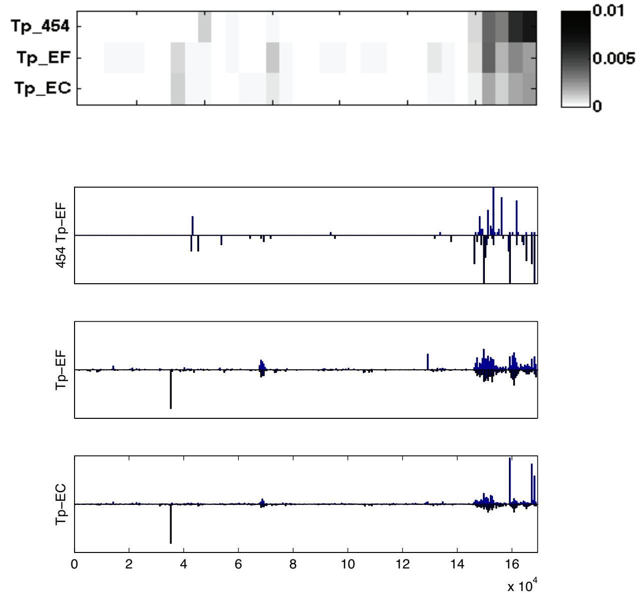

chr_17

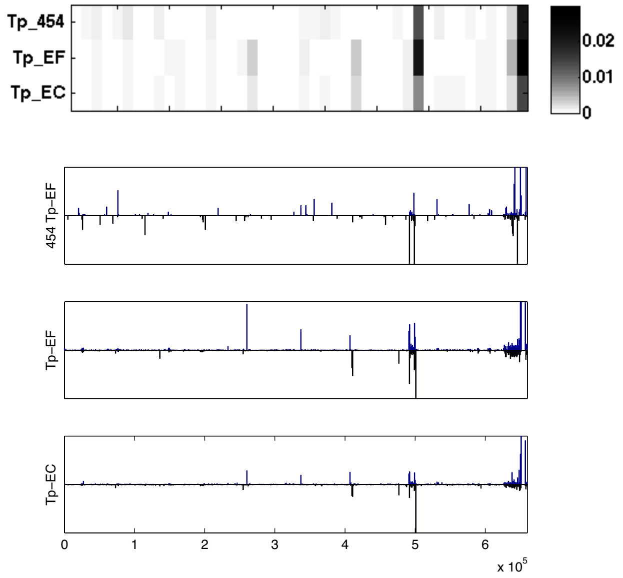

chr_18

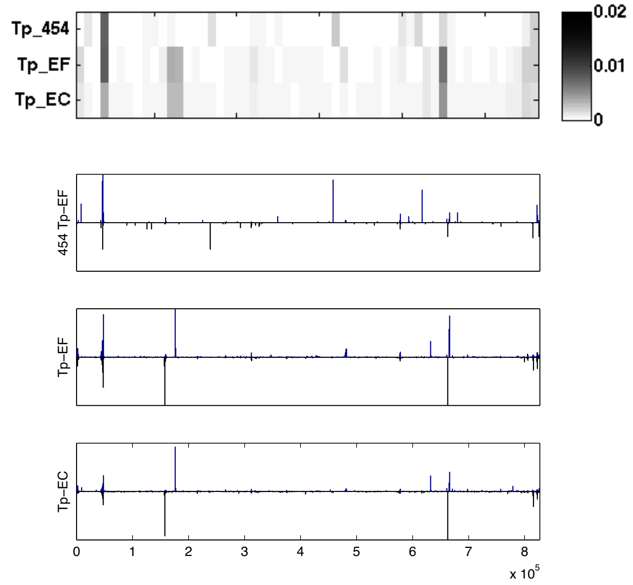

chr_19a_19

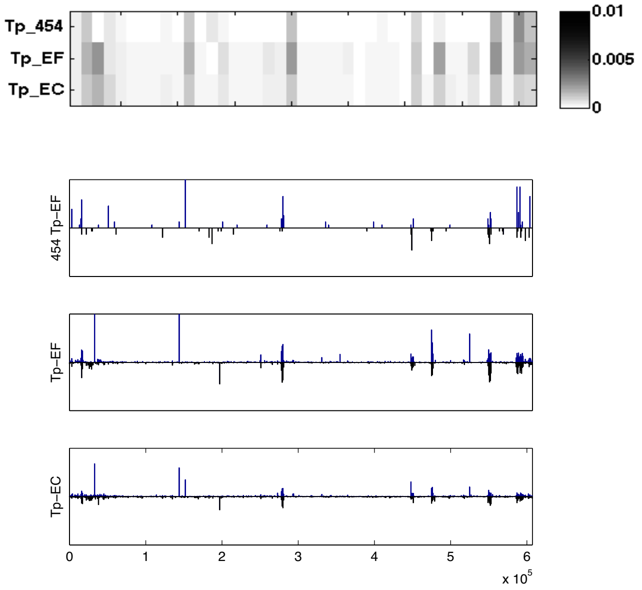

chr_19b_31

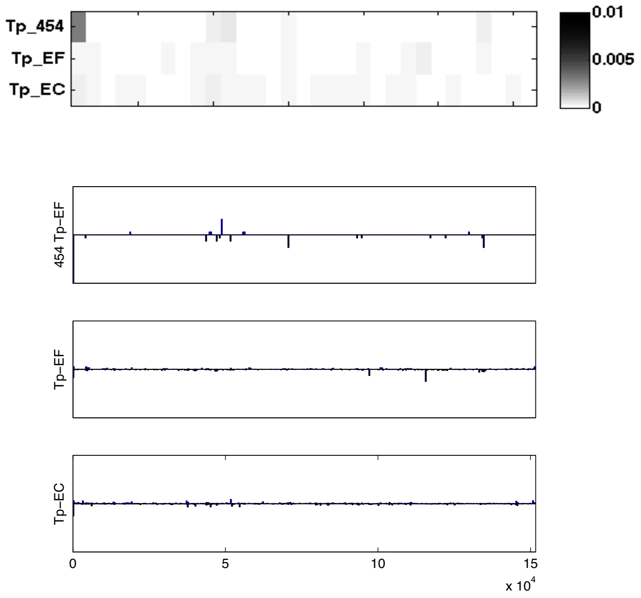

chr_19c_29

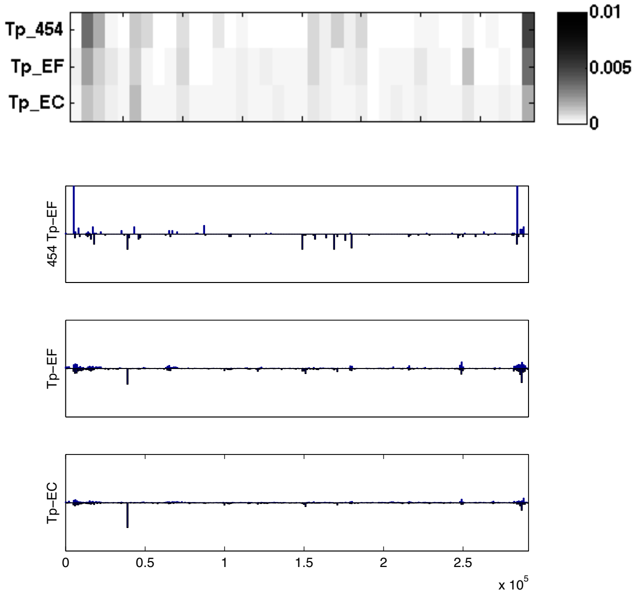

chr_20

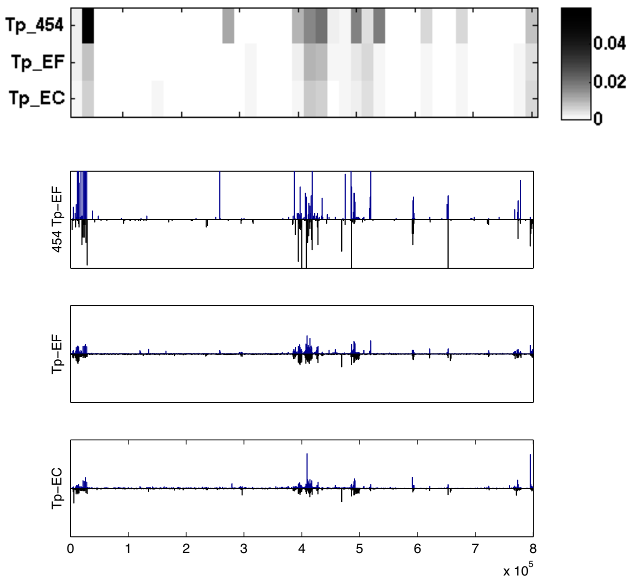

chr_22

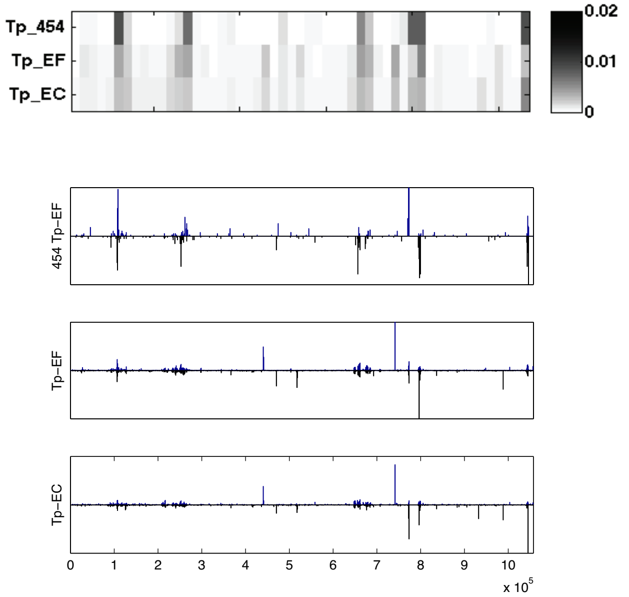

chr_23

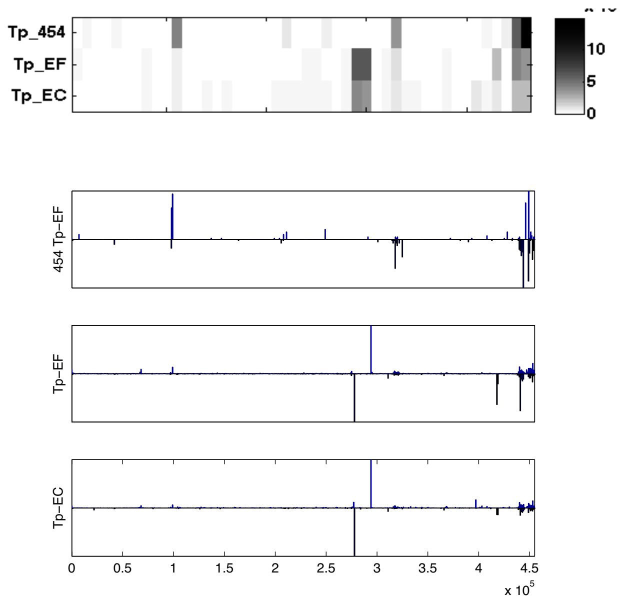

chr_24

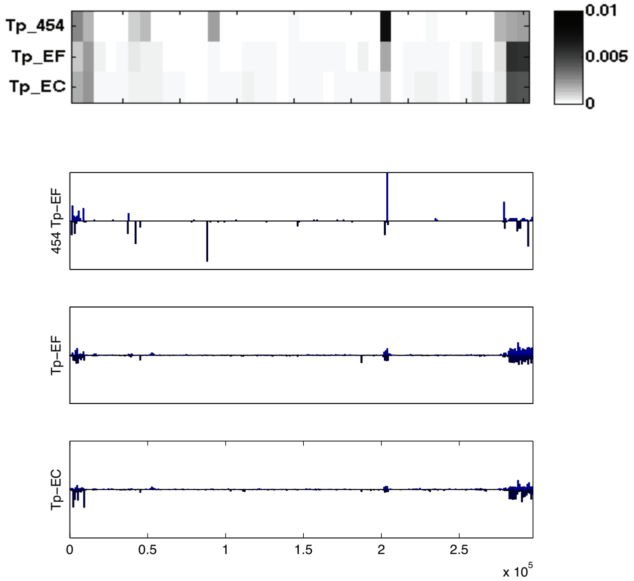

Supplement: Figure S3 — Heatmaps and histograms of small RNA candidate abundance mapped along all of the T. pseudonana chromosomes. (PDF) [file pone.0022870.s003.pdf]
