# Supplementary figures and images for: Characterization of the Small RNA Transcriptome of the Diatom, Thalassiosira pseudonana
Source: PLoS One. 2011 Aug 12;6(8):e22870. doi: 10.1371/journal.pone.0022870 (PMC3155517; doi:10.1371/journal.pone.0022870)

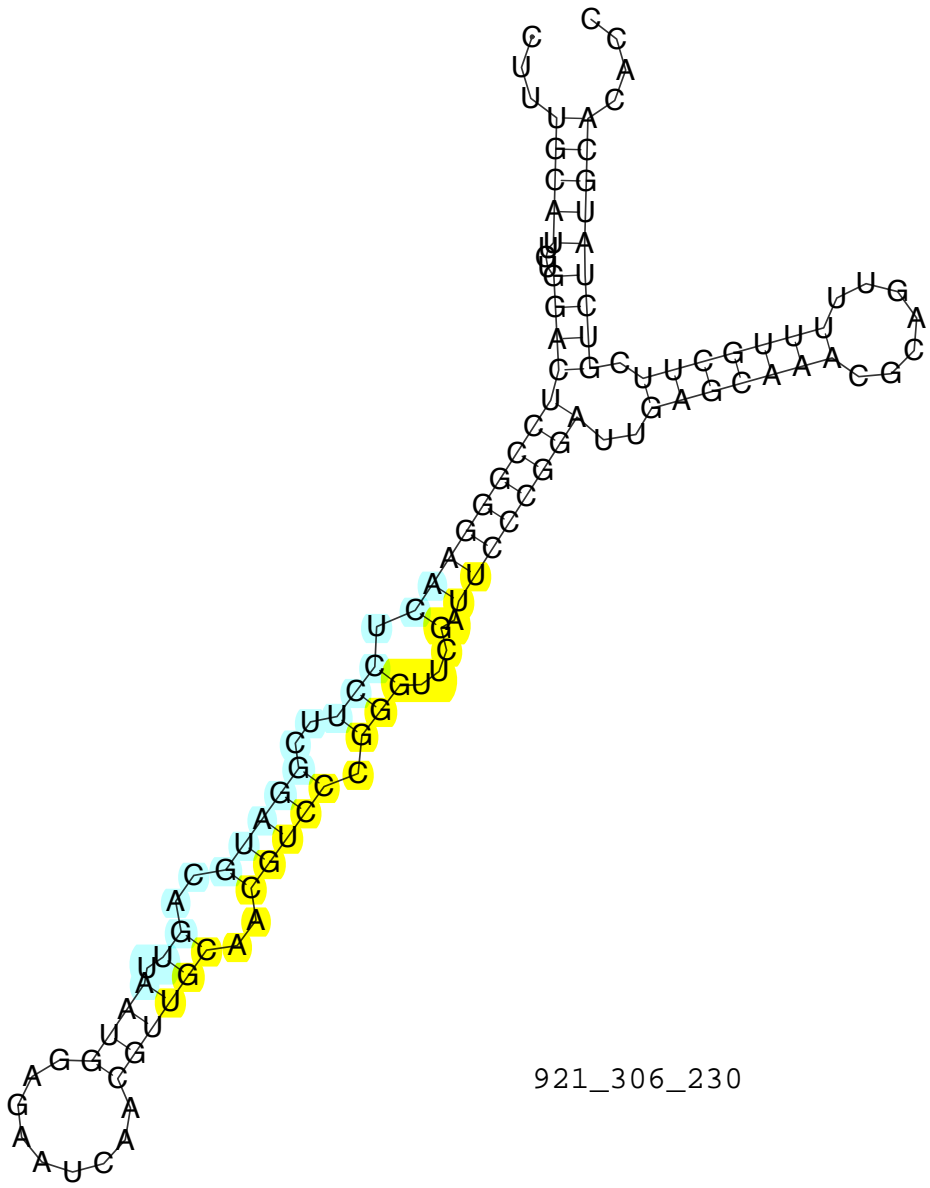

921\_306\_230

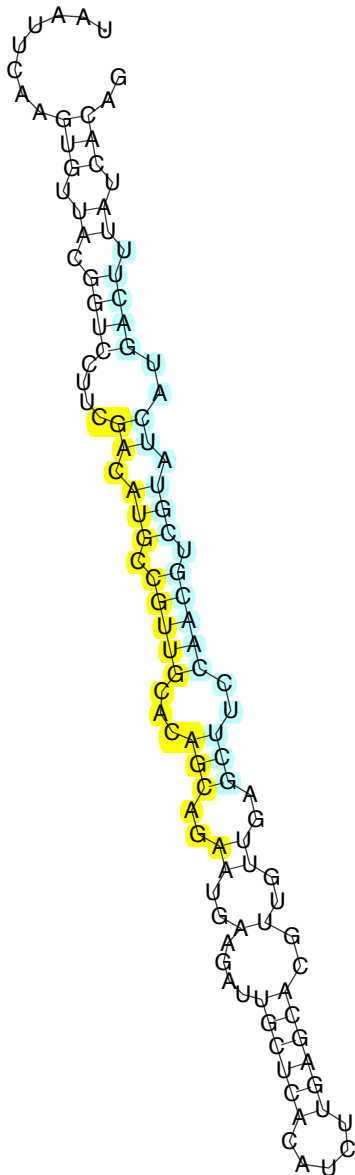

1072\_1906\_853



1177\_1978\_318

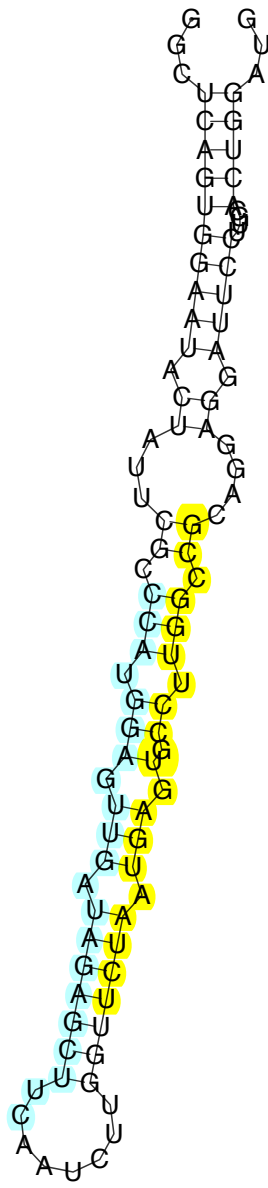

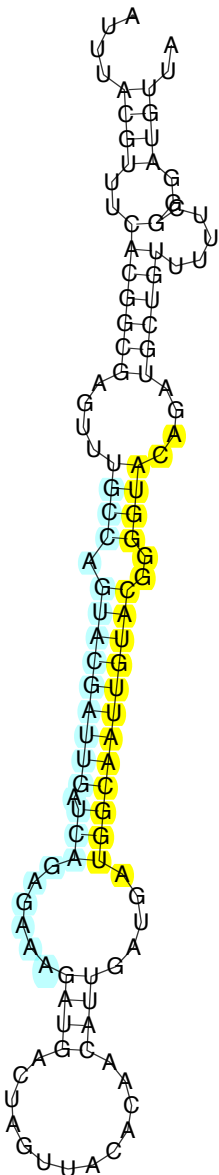

1180\_1482\_783

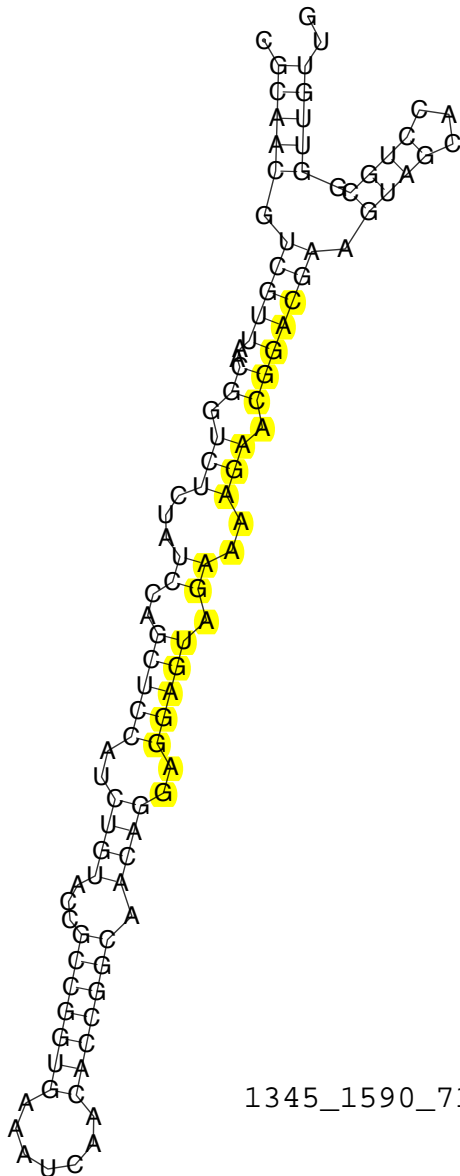

1345\_1590\_717

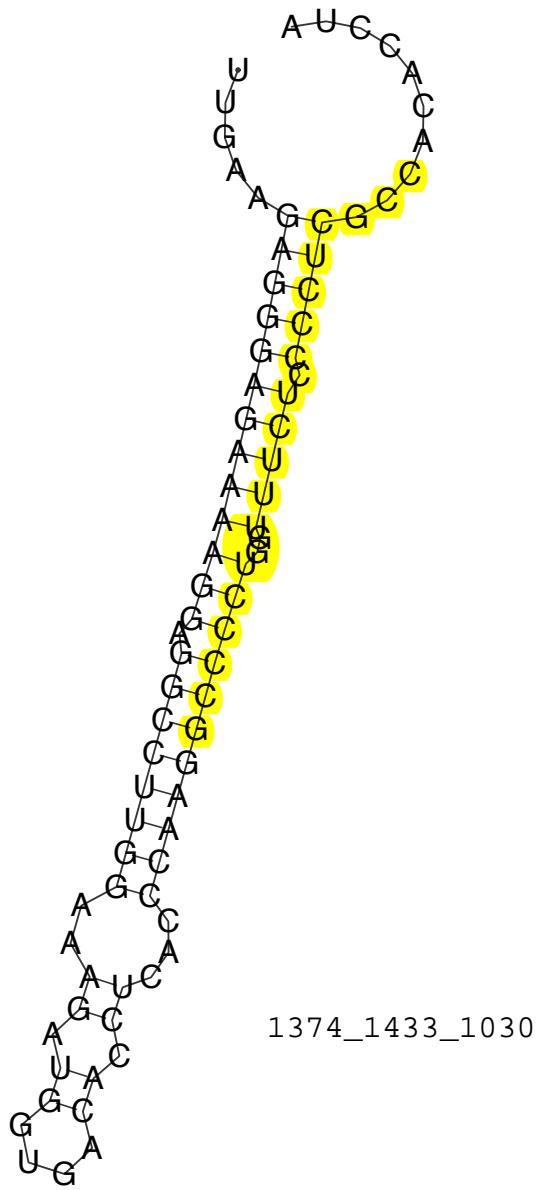

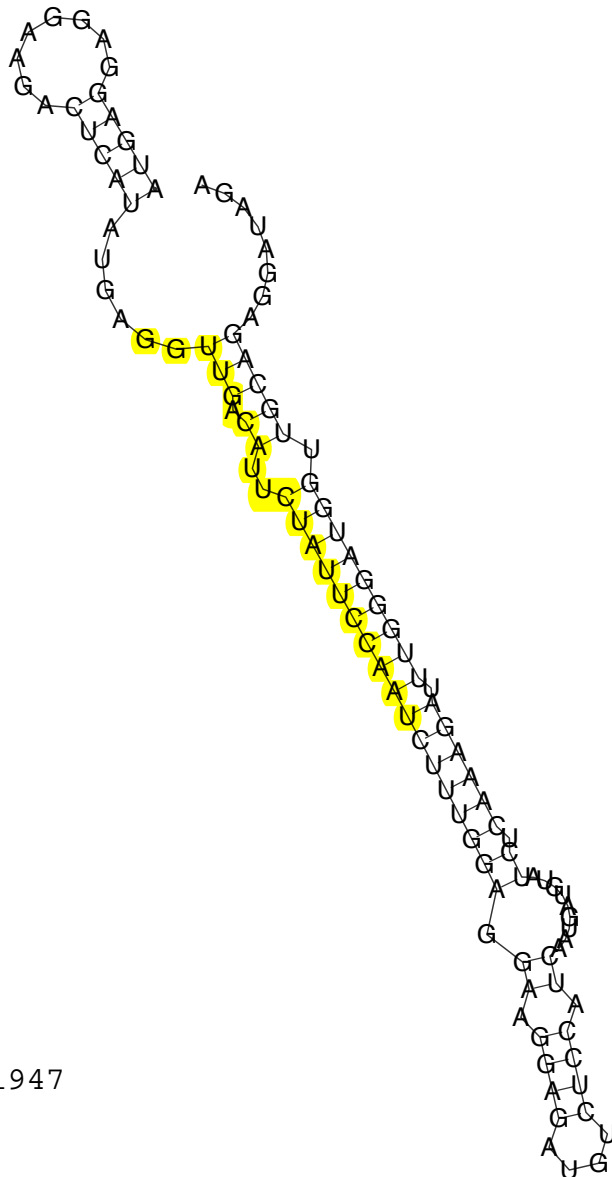

1381\_423\_1947

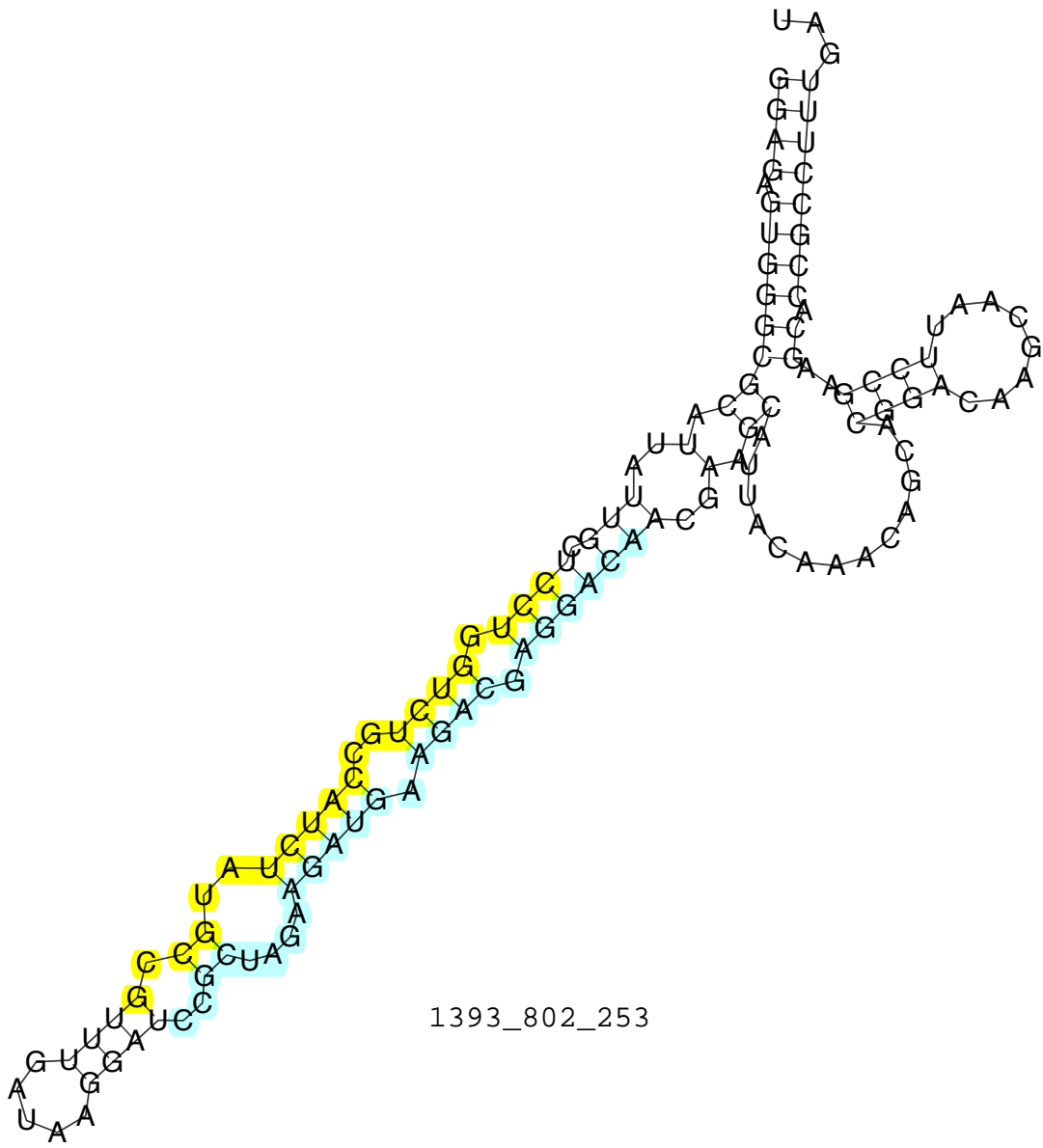

1393\_802\_253

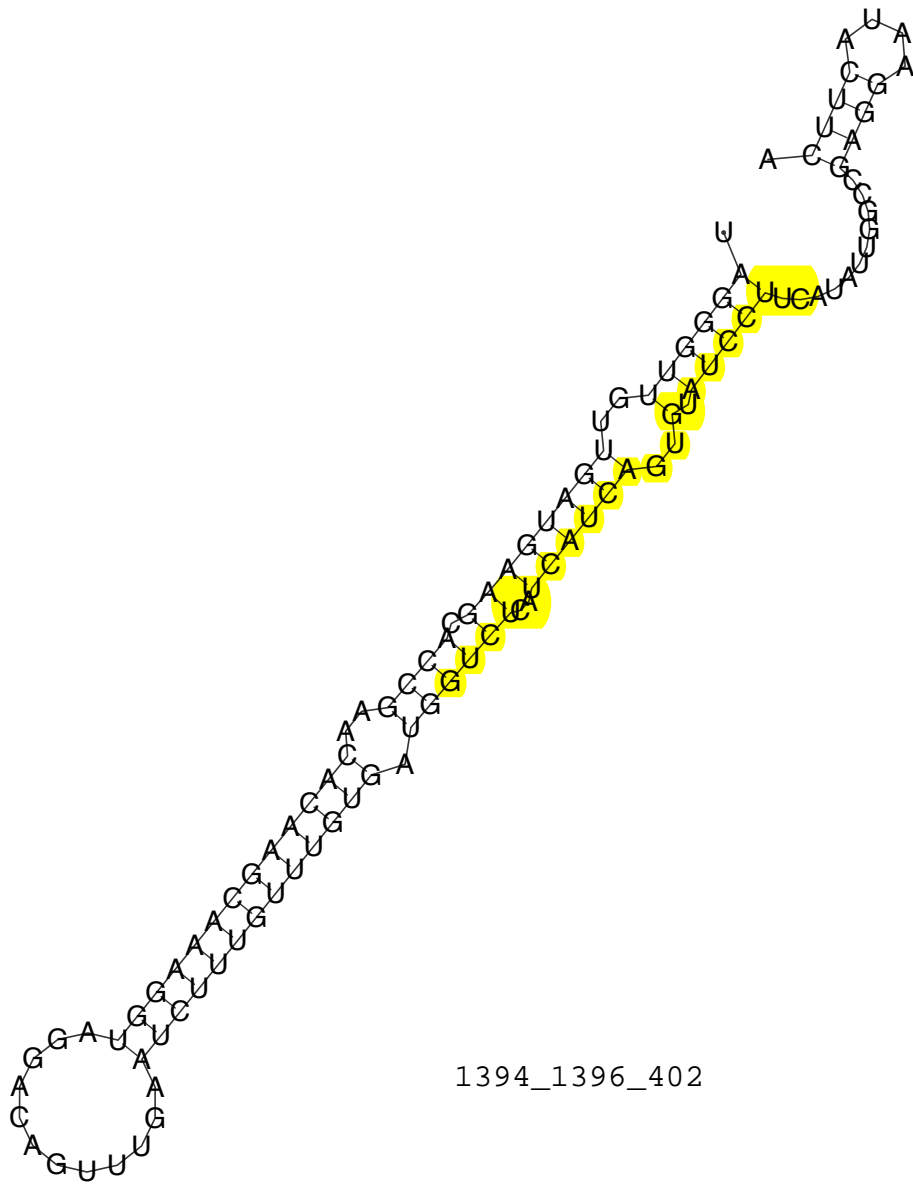

1394\_1396\_402

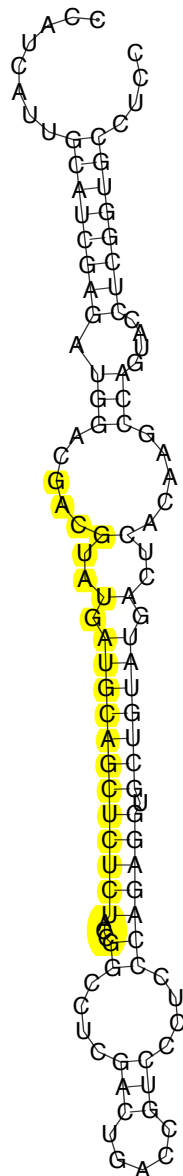

1431\_1377\_1517

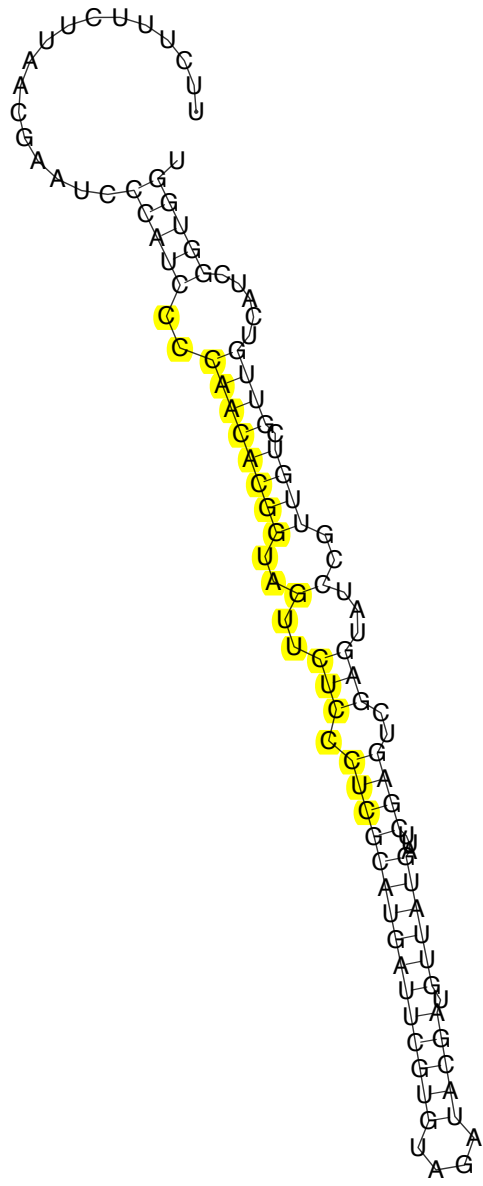

1442\_152\_704

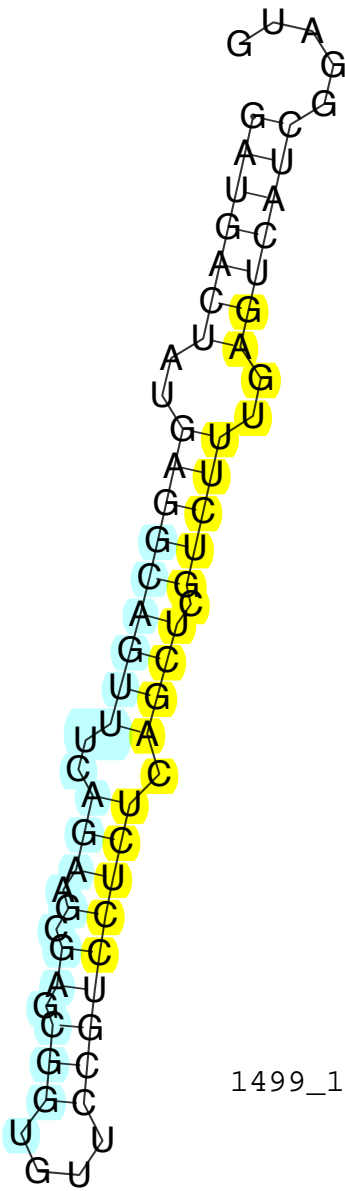

1499\_1964\_1108

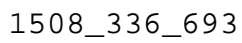



1567\_164\_827

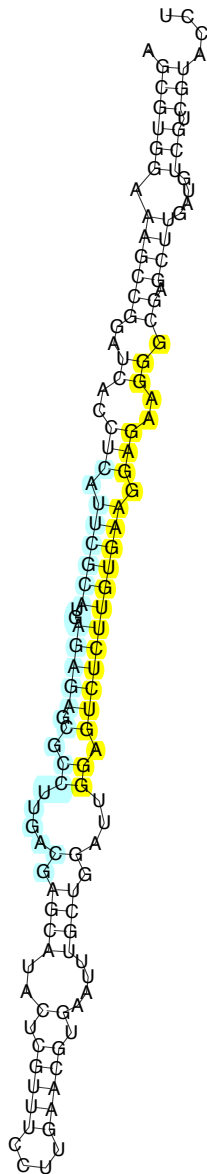

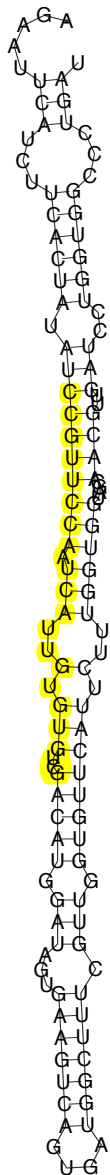

1233\_1805\_1228



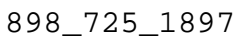

898\_725\_1897

906\_1339\_822

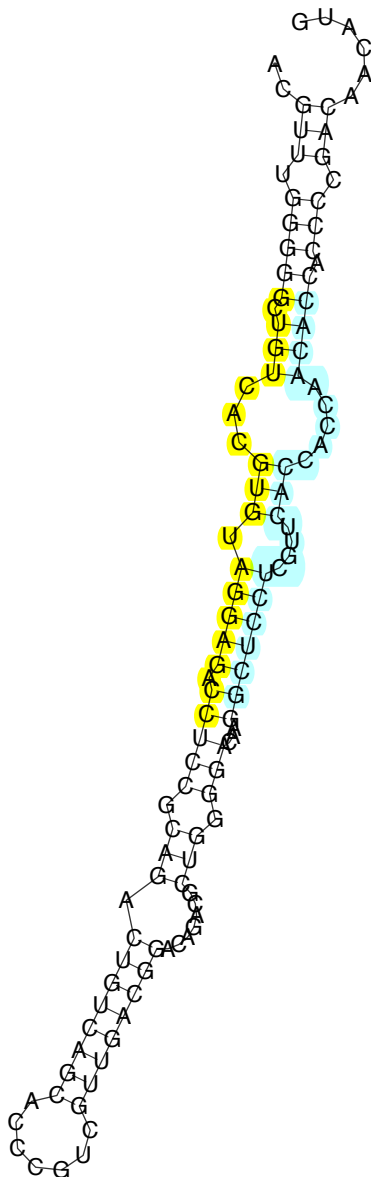







1408\_1985\_285

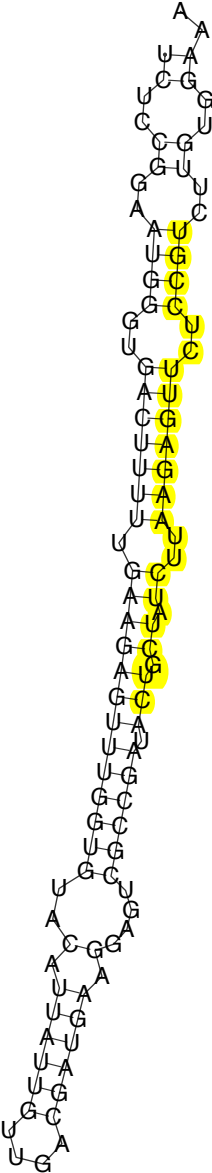

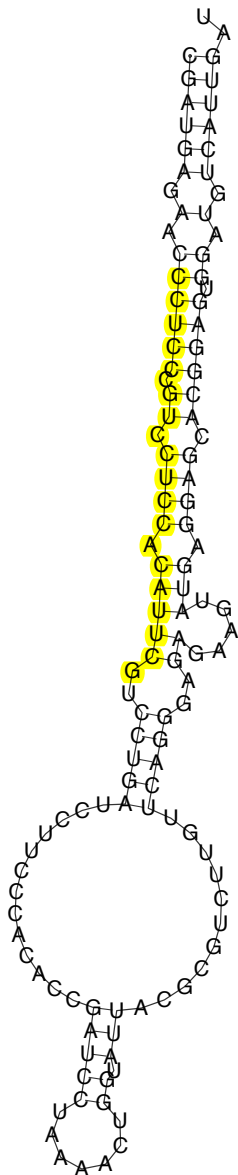

1427\_942\_1564

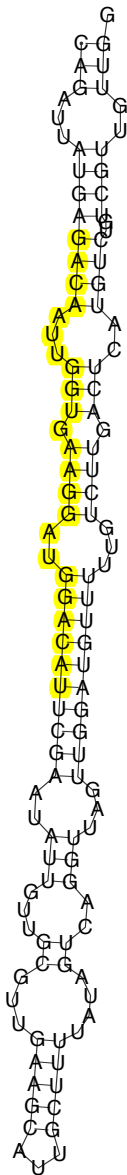

1476\_61\_688

1595\_1216\_233

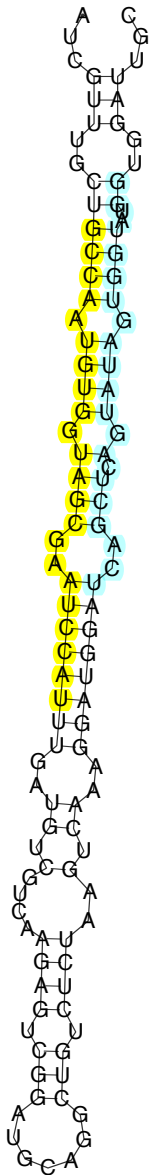

Supplement: Figure S4 — Predicted secondary structures of the microRNA candidates in the T. pseudonana small RNA libraries. The precursor sequences have been folded with RNAfold. The mature miRNA portions are highlighted in yellow. The miRNA* arm, if present, is highlighted in blue. (PDF) [file pone.0022870.s004.pdf]
