# Supplementary material for: Characterization of the Small RNA Transcriptome of the Diatom, Thalassiosira pseudonana
Source: PLoS One. 2011 Aug 12;6(8):e22870. doi: 10.1371/journal.pone.0022870 (PMC3155517; doi:10.1371/journal.pone.0022870)
Supplement: Text S1 — List of proteins and motifs used in search for RNAi machinery in T. pseudonana genome. (DOC) [file pone.0022870.s008.doc]

**Additional File 1 – List of proteins and motifs used in search for presence of RNAi machinery in the *T. pseudonana* genome**

|  |  |
| --- | --- |
| **RNAi-related** | **Protein motifs (Pfam ID)** |
| **proteins** |  |
|  |  |
| **Argonaute** |  |
|  | PAZ (PF02170) |
|  | PIWI (PF02171) |
|  |  |
| **Armitage** |  |
|  |  |
| **Aubergine** | PAZ and PIWI (PF02170, PF02171) |
|  |  |
| **chp1** | Chromo (PF00385) |
|  |  |
| **Dicer, Dicer-like** |  |
|  | DUF283 or dsRNA_bind (PF03368) |
|  | Helicase_C (PF00271) |
|  | Ribonuclease_3 (PF00636) |
|  | DEAD (PF00270) |
|  | dsrm (PF00035) |
|  | PAZ (PF02170) |
|  |  |
| **ddm1** |  |
|  | SFN2_N (PF00176) |
|  | Helicase_C (PF00271) |
|  |  |
| **dead** |  |
|  | DEAD (PF00270) |
|  | Helicase_C (PF00271) |
|  |  |
| **DExDc** |  |
| **(grouped with HELICc)** | DEAD (PF00270) |
|  | Helicase_C (PF00271) |
|  |  |
| **DExH** |  |
|  | HA2 (PF04408) |
|  | DEAD (PF00270) |
|  | Helicase_C (PF00271) |
|  |  |
| **DGCR8** |  |
|  | WW (PF00397) |
|  | dsrm (PF00035) |
|  |  |
| **Drosha** |  |
|  | dsrm (PF00035) |
|  | Ribonuclease_3 (PF00636) |
|  |  |
| **DSRM** |  |
| **ds RNA binding motif,** | dsrm (PF00035) |
| **Ribonuclease III, Rnase III** | Ribonuclease_3 (PF00636) |
|  |  |
| **dsRNA binding** | dsrm (PF00035) |
|  |  |
| **HDAC6** |  |
|  | Hist_deacetyl (PF00850) |
| **Histone deacetylase 6** | zf-UBP (PF02148) |
|  |  |
| **Hua Enhancer (HEN1)** |  |
|  |  |
| **Loquacious** |  |
|  | dsrm (PF00035) |
|  |  |
| **MILI** |  |
| **Piwi-like protein 2,** | PAZ (PF02170) |
| **Piwil2, Mili** | PIWI (PF02171) |
|  |  |
| **MIWI** |  |
| **Piwi-like protein 1,** | PAZ (PF02170) |
| **Piwil1, Miwi** | PIWI (PF02171) |
|  |  |
| **Pasha** |  |
|  | dsrm (PF00035) |
|  |  |
| **Paz** |  |
|  | PAZ (PF02170) |
|  | PIWI (PF02171) |
|  |  |
| **Piwi** |  |
|  | PAZ (PF02170) |
|  | PIWI (PF02171) |
|  |  |
| **R2D2** |  |
|  | dsrm (PF00035) |
|  |  |
|  |  |
| **RDR1** |  |
|  | Fungal_trans (PF04082) |
| **RNA-dependent RNA** | Zn_clus (PF00172) |
| **polymerase** | RdRP (PF05183) |
|  |  |
| **RDR2** |  |
|  |  |
| **Ribonuclease III** |  |
|  | Ribonuclease_3 (PF00636) |
|  |  |
| **RdRP** | RdRP_3 (PF00998) |
|  |  |
| **RNA helicase** |  |
|  | DEAD (PF00270) |
|  | Helicase_C (PF00271) |
|  |  |
| **Serrate** | none |
|  |  |
| **Slicer** |  |
|  | PAZ (PF02170) |
|  | PIWI (PF02171) |
|  |  |
| **Tas3 / RITS** | none |
|  |  |
| **Twi1** |  |
|  | PF00201, UDPGT |
|  | PAZ (PF02170) |
|  | PIWI (PF02171) |
|  |  |
| **Zwille** |  |
|  | PAZ (PF02170) |
|  | PIWI (PF02171) |
|  |  |
